# A Review of CYP3A Drug-Drug Interaction Studies: Practical Guidelines for Patients Using Targeted Oral Anticancer Drugs

**DOI:** 10.3389/fphar.2021.670862

**Published:** 2021-08-30

**Authors:** Laura Molenaar-Kuijsten, Dorieke E. M. Van Balen, Jos H. Beijnen, Neeltje Steeghs, Alwin D. R. Huitema

**Affiliations:** ^1^ Department of Pharmacy & Pharmacology, The Netherlands Cancer Institute—Antoni van Leeuwenhoek, Amsterdam, Netherlands; ^2^ Department of Pharmaceutical Sciences, Utrecht University, Utrecht, Netherlands; ^3^ Department of Medical Oncology and Clinical Pharmacology, The Netherlands Cancer Institute—Antoni van Leeuwenhoek, Amsterdam, Netherlands; ^4^ Department of Clinical Pharmacy, University Medical Center Utrecht, Utrecht University, Utrecht, Netherlands; ^5^ Department of Pharmacology, Princess Máxima Center for Pediatric Oncology, Utrecht, Netherlands

**Keywords:** drug interaction, cytochrome P450 enzyme, CYP3A inhibitor, CYP3A inducer, cancer, kinase inhibitor

## Abstract

Many oral anticancer drugs are metabolized by CYP3A. Clinical drug-drug interaction (DDI) studies often only examine the effect of strong CYP3A inhibitors and inducers. The effect of moderate or weak inhibitors or inducers can be examined using physiologically based pharmacokinetic simulations, but data from these simulations are not always available early after approval of a drug. In this review we provide recommendations for clinical practice on how to deal with DDIs of oral anticancer drugs if only data from strong CYP3A inhibitors or inducers is available. These recommendations were based on reviewed data of oral anticancer drugs primarily metabolized by CYP3A and approved for the treatment of solid tumors from January 1st, 2013 to December 31st, 2015. In addition, three drugs that were registered before the new EMA guideline was issued (i.e., everolimus, imatinib, and sunitinib), were reviewed. DDIs are often complex, but if no data is available from moderate CYP3A inhibitors/inducers, a change in exposure of 50% compared with strong inhibitors/inducers can be assumed. No *a priori* dose adaptations are indicated for weak inhibitors/inducers, because their interacting effect is small. In case pharmacologically active metabolites are involved, the metabolic pathway, the ratio of the parent to the metabolites, and the potency of the metabolites should be taken into account.

## Introduction

Oral targeted anticancer drugs are important drugs for the treatment of cancer. Most oral anticancer drugs are metabolized by CYP3A; therefore, patients are at risk for drug-drug interactions (DDI). Because many of these drugs show an exposure-efficacy and an exposure-toxicity relationship, a change in exposure to these drugs can be highly relevant (Verheijen et al., 2017; Groenland et al., 2019). This change in exposure as a consequence of a DDI could result in adverse events if exposure is increased, or treatment failure if exposure is decreased (in case of prodrugs vice versa).

DDI studies are performed before registration of a drug, based on the metabolism of the drug and following the recommendations of the EMA and FDA (Food and Drug Administration. Center for Drug Evaluation and Research, 2009; Food and Drug Administration. Center for Drug Evaluation and Research, 2020a; European Medicines Agency Committee for Medicinal Products For Human Use (CHMP), 2014a). These studies use strong CYP3A inhibitors (e.g., itraconazole or ketoconazole) and inducers (e.g., rifampin) since the guidelines of the EMA and FDA advise a worst-case approach. Subsequently, the effects of moderate and weak inhibitors or inducers are extrapolated from these data using physiologically based pharmacokinetic (PBPK) simulations (European Medicines Agency Committee for Medicinal Products For Human Use (CHMP), 2015a; Food and Drug Administration. Center for Drug Evaluation and Research, 2020a). In short, conducting a PBPK simulation consists of three steps: model development, model verification, and model application. First a physiologically based model is built for the substrate and interacting drug (for the latter also the SimCYP library can be used), including for example PK data. Secondly, the models are verified, e.g., by simulating a concentration-time profile and comparing it with the data from clinical studies. Subsequently, the two models are linked and drug-drug interactions can be simulated. Before the effects of moderate and weak inhibitors and inducers can be predicted, first the models should be verified using data from clinical DDI studies with strong inhibitors and inducers. The use of PBPK models is described in several guidelines of the FDA (Food and Drug Administration. Center for Drug Evaluation and Research, 2018; Food and Drug Administration. Center for Drug Evaluation and Research, 2020a; Food and Drug Administration. Center for Drug Evaluation and Research, 2020b). There is, however, a critical problem with the above described DDI studies performed before drug approval. Despite the fact that moderate and weak inhibitors and inducers are far more frequently used than the strong CYP3A inhibitors and inducers, clinical data on moderate and weak inhibitors and inducers is often lacking. This problem is partly overcome by the, increasingly performed, PBPK simulations. But, data from these PBPK simulations are not always available early after approval of a drug. This is for example the case for drugs that are conditionally approved, as is the case for, for instance, larotrectinib, and lorlatinib (Food and Drug Administration, 2017; Food and Drug Administration, 2018; Chen et al., 2020; Patel et al., 2020).

To determine which drugs might influence the metabolism of oral anticancer drugs, the Flockhart Table can be consulted (Flockhart, 2007). The Flockhart Table displays drugs that inhibit or induce specific CYP enzymes, for example CYP3A (Flockhart, 2007). The interacting drugs are placed in groups according to the inhibition or induction capacity, and are classified in broad ranges. Weak inhibitors increase the AUC by ≥1.25–<2-fold, moderate inhibitors by ≥2–<5-fold, and strong inhibitors by ≥5-fold (Flockhart, 2007; Food and Drug Administration. Center for Drug Evaluation and Research, 2020a). Weak inducers decrease the AUC by ≥20–<50%, moderate inducers by ≥50–<80%, and strong inducers by ≥80% (Food and Drug Administration, 2020a).

The aim of this review was to provide recommendations for clinical practice on how to deal with DDIs of oral anticancer drugs if only data from strong CYP3A inhibitors or inducers is available. To achieve this goal, we compared results from DDI studies with strong inhibitors or inducers with results with moderate or weak inhibitors or inducers, to extrapolate results to clinical practice and formulate an advice on how to deal with DDIs for which data is lacking.

## Methods

**FIGURE 1:**
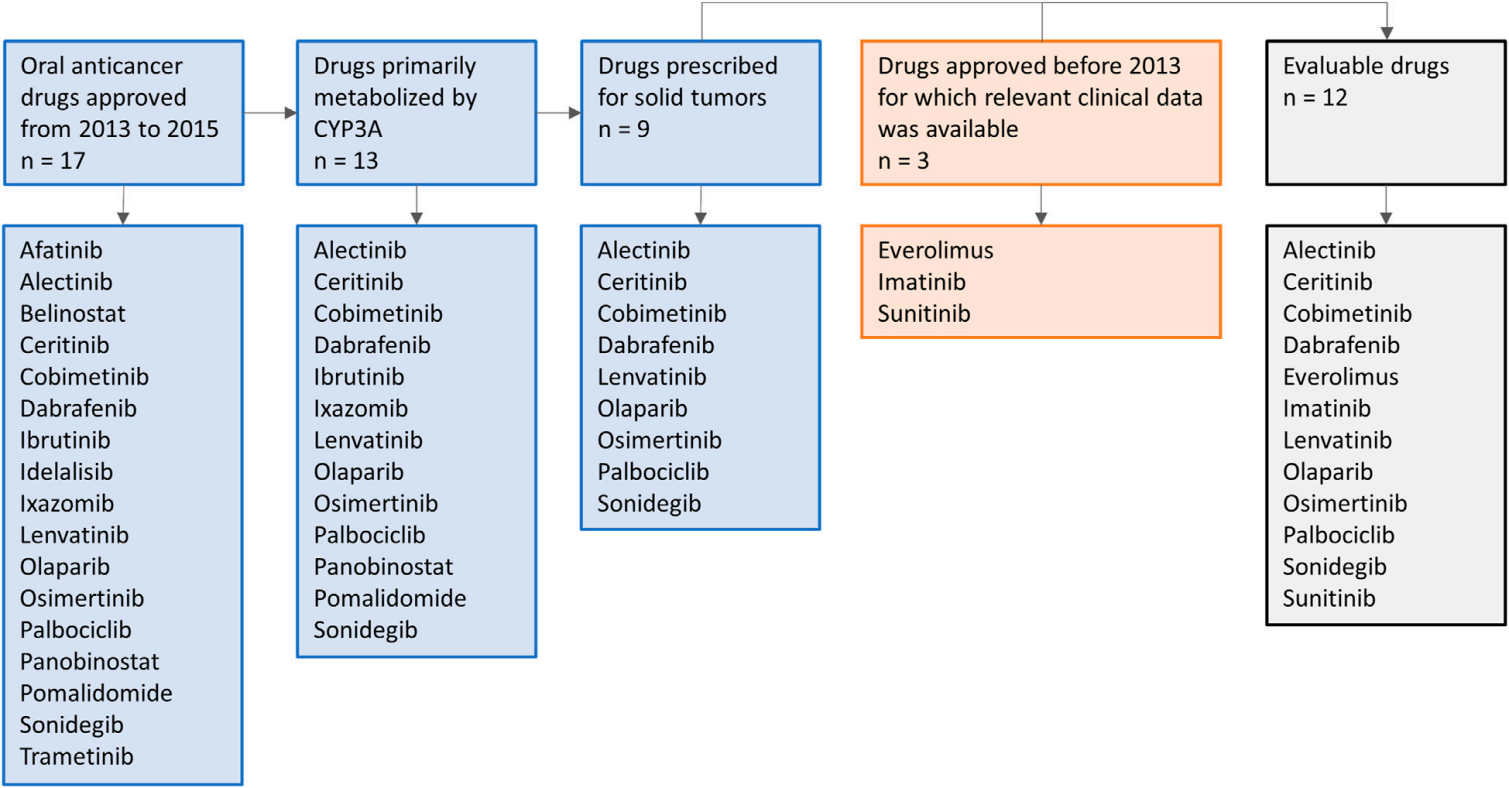
Schematic overview of drug selection.

Oral anticancer drugs, used for the treatment of solid tumors, were selected based on their metabolism and year of approval. On January 1st, 2013, the EMA guideline on the investigation of DDIs came into effect (European Medicines Agency Committee for Medicinal Products For Human Use (CHMP), 2015b). To allow several years of follow-up after approval, in which clinical DDI studies with these drugs might be conducted, an inclusion cut-off in December 2015 was chosen. Therefore, all drugs primarily metabolized by CYP3A and approved for the treatment of solid tumors from January 1st, 2013 to December 31st, 2015 were selected. In addition, we included three drugs that were registered before the new EMA guideline was issued (i.e., everolimus, imatinib, and sunitinib), to illustrate how DDI studies were performed with the prior guideline. An overview of the drug selection is shown in Figure 1. Firstly, the US FDA Clinical Pharmacology and Biopharmaceutics Review and the Summary of Product Characteristics of these drugs were studied for data on DDI studies. Second, PubMed was searched using the search terms (drug name) AND [drug-drug interaction (study)] OR (drug name of most used potent inhibitor and inducer). Furthermore, citation snowballing was used to find other articles of interest. The articles, including case reports, in which no AUCs were reported, or in which the dose of the victim drug was different between the control group and group with combination treatment, and *in vitro* studies were excluded. We searched the articles for the change in AUC (preferably the AUC_0–∞_) of the victim drug in combination with the studied CYP3A inhibitor or inducer, compared with administration of the victim drug alone. We visualized this by making graphs using the ratios of adjusted means of the combination versus the victim drug alone, whereby the victim drug alone was rated as 100% exposure. The studied inhibitors and inducers were grouped according to their interaction potential, which was reported in the reviewed articles and checked with the Flockhart Table (Flockhart, 2007).

## Results

Table 1 gives a summary of the DDI studies of the twelve selected oral anticancer drugs. In Table 2 a detailed overview of the results is shown. The results are described for the drugs without active metabolites first and for the drugs with active metabolites thereafter.

**TABLE 1:** Summary table of the results of DDI studies performed with the reviewed oral oncolytic drugs.

Drug	Effect CYP3A inhibitors^a^			Effect CYP3A inducers^a^		
	Strong	Moderate	Weak	Strong	Moderate	Weak
Alectinib^b^	36% ↑			18.4% ↓		
Ceritinib	118.5% ↑ (51–186)	37% ↑		68.5% ↓ (67–70)	43% ↓	
Cobimetinib	572% ↑	280.5% ↑ (226–335)	3% ↑	83% ↓	72% ↓	13% ↓
Dabrafenib	71% ↑			34% ↓		
• Hydroxy-dabrafenib	82% ↑			30% ↓		
• Desmethyl-dabrafenib	68% ↑			73% ↑		
• Carboxy-dabrafenib	16% ↓					
Everolimus	1,430% ↑	220% ↑ (74–340)		63% ↓		
Imatinib	18.5% ↑ (−3.1–40.1)			73.3% ↓ (72.5–74)		37.1% ↓ (30.2–44)
• N-desmethyl-imatinib	16.75% ↑ (−5–38.5)			10.8% ↓ (9.8–11.7)		4.1% ↑
Lenvatinib	14.5% ↑			6.2% ↑ (−18.2–30.6)		
Olaparib	161% ↑ (152–170)	115% ↑ (98–126)	1.5% ↑ (1–2)	79% ↓ (71–87)	57.3% ↓ (53–60)	0%↓
Osimertinib	24.2% ↑			78.5% ↓	42% ↓	0%↓
Palbociclib	86.8% ↑	40% ↑ (38–42)	0.4% ↑ (0.3–0.4)	85.2% ↓	35% ↓ (32–38)	
Sonidegib	122.8% ↑ (42–253)	98% ↑ (36–179)		76.6% ↓ (66–88)	49% ↓ (29–65)	
Sunitinib^c^	51% ↑	11% ↑		46% ↓		

a Reported as percentage of AUC change, if multiple DDI studies were performed the mean AUC change and range are reported.

b Sum of alectinib and M4.

c Sum of sunitinib and SU12662, except for the moderate inhibitor.

**TABLE 2:** Detailed overview of the results of DDI studies performed with the reviewed oral oncolytic drugs.

Drug (year of market approval)	(Primary) metabolism	Target Verheijen et al. (2017)	Inter-patient variability (%CV)	Dose-linearity	DDI study with (interaction potential)	Change in AUC	Recommendations Summary of product Characteristics	Type of trial	References
Alectinib (2015)	CYP3A	ALK	46%	Dose proportional exposure	Posaconazole (strong CYP3A inhibitor)	AUC_0–∞_ 75% ↑ (90% CI 57–95)	Be careful when combining alectinib with strong inhibitors of CYP3A	Clinical trial	Food and Drug Administration (2015a) and Morcos et al. (2017)
						M4 AUC_0–∞_ 24.9% ↓ (90% CI 12.3–35.6)			
						Sum alectinib and M4 AUC_0–∞_ 36% ↑ (90% CI 24–49)			
					Rifampin (strong CYP3A inducer)	AUC_0–∞_ 73.2% ↓ (90% CI 69.9–76.2)	Be careful when combining alectinib with strong inducers of CYP3A	Clinical trial	Food and Drug Administration (2015a) and Morcos et al. (2017)
						M4 AUC_0-∞_ 79% ↑ (90% CI 58–102)			
						Sum alectinib and M4 AUC_0–∞_ 18.4% ↓ (90% CI 9.9–26)			
Ceritinib (2014)	CYP3A	ALK	74%	Nonlinear PK	Ketoconazole (strong CYP3A inhibitor)	Single dose AUC_0–∞_ 186% ↑ (90% CI 146–233)	Avoid coadministration of strong CYP3A inhibitors or reduce the dose of ceritinib to 150 mg QD	Clinical trial	Food and Drug Administration (2014a)
						Steady–state AUC 51% ↑ (90% CI 43–59)		PBPK simulation	
					Fluconazole (moderate CYP3A inhibitor)	AUC 37% ↑ (90% CI 31–42)		PBPK simulation	Food and Drug Administration (2014a)
					Rifampin (strong CYP3A inducer)	Single dose AUC_0–∞_ 70% ↓ (90% CI 61–77)	Avoid coadministration of strong CYP3A inducers	Clinical trial	Food and Drug Administration (2014a)
						Steady-state AUC 67% ↓ (90% CI 64–70)		PBPK simulation	
					Efavirenz (moderate CYP3A inducer)	AUC 43% ↓ (90% CI 38–48)		PBPK simulation	Food and Drug Administration (2014a)
Cobimetinib (2015)	CYP3A	MEK	61%	Dose proportional exposure	Itraconazole (strong CYP3A inhibitor)	AUC_0-∞_ 572% ↑ (90% CI 464–702)	Avoid coadministration of strong and moderate CYP3A inhibitors or reduce the dose of cobimetinib to 20 mg QD (short term use)	Clinical trial	Food and Drug Administration (2014b)
					Erythromycin (moderate CYP3A inhibitor)	AUC 335% ↑		PBPK simulation	Food and Drug Administration (2014b) and Budha et al. (2016)
					Diltiazem (moderate CYP3A inhibitor)	AUC 226% ↑		PBPK simulation	Food and Drug Administration (2014b) and Budha et al. (2016)
					Fluvoxamine (weak CYP3A inhibitor)	AUC 3% ↑		PBPK simulation	Food and Drug Administration (2014b) and Budha et al. (2016)
					Rifampin (strong CYP3A inducer)	AUC 83% ↓	Avoid coadministration of strong and moderate CYP3A inducers	PBPK simulation	Food and Drug Administration (2014b) and Budha et al. (2016)
					Efavirenz (moderate CYP3A inducer)	AUC 72% ↓		PBPK simulation	Food and Drug Administration (2014b) and Budha et al. (2016)
					Vemurafenib (weak CYP3A inducer)	AUC_0–24h_ 13% ↓		Clinical trial	Food and Drug Administration (2014b)
Dabrafenib (2013)	CYP2C8/CYP3A	BRAF	38%	Dose proportional exposure at single dose, but less than dose-proportional after repeat twice daily dosing (likely due to auto-induction)	Ketoconazole (strong CYP3A inhibitor)	AUC_0–12h_ 71% ↑ (90% CI 55–90)	Be careful when combining dabrafenib with strong inhibitors of CYP3A	Clinical trial	Food and Drug Administration (2012), Suttle et al. (2015), and European Medicines Agency Committee for Medicinal Products For Human Use (CHMP) (2018)
						Hydroxy-dabrafenib AUC_0–12h_ 82% ↑ (90% CI 61–105)			
						Desmethyl-dabrafenib AUC_0–12h_ 68% ↑ (90% CI 47–93)			
						Carboxy-dabrafenib AUC_0–12h_ 16% ↓ (90% CI 4–27)			
					Rifampin (strong CYP3A inducer)	AUC 34% ↓	Avoid coadministration of CYP3A inducers	Clinical trial	European Medicines Agency Committee for Medicinal Products For Human Use (CHMP) (2018)
						Desmethyl-dabrafenib AUC 30% ↓			
						Carboxy-dabrafenib AUC 73% ↑			
Everolimus (2003)	CYP3A/P-gp	mTOR	36%	Dose proportional exposure	Ketoconazole (strong CYP3A inhibitor)	AUC_0–∞_ 1,430% ↑ (90% CI 1020–2,150)	Avoid coadministration of strong CYP3A inhibitors. Avoid coadministration of moderate CYP3A inhibitors or reduce the dose of everolimus to 2.5 or 5 mg QD	Clinical trial	Kovarik et al. (2005b) and Food and Drug Administration (2008)
					Erythromycin (moderate CYP3A inhibitor)	AUC_0–∞_ 340% ↑ (90% CI 250–440)		Clinical trial	(European Medicines Agency Committee for Medicinal Products For Human Use (CHMP); Kovarik et al. (2005c) and Food and Drug Administration (2008)
					Verapamil (moderate CYP3A inhibitor)	AUC_0–∞_ 250% ↑ (90% CI 210–290)		Clinical trial	(European Medicines Agency Committee for Medicinal Products For Human Use (CHMP); Kovarik et al., (2005a) and Food and Drug Administration (2008)
Everolimus (2003)	CYP3A/P-gp	mTOR	36%	Dose proportional exposure	Imatinib (moderate CYP3A inhibitor)	AUC 270% ↑	Avoid coadministration of strong CYP3A inhibitors. Avoid coadministration of moderate CYP3A inhibitors or reduce the dose of everolimus to 2.5 or 5 mg QD	Clinical trial	(European Medicines Agency Committee for Medicinal Products For Human Use (CHMP), 2006)
					Cyclosporine (moderate CYP3A inhibitor)	Neoral^®^ AUC_0–∞_ 168% ↑ (90% CI 122–224)		Clinical trial	Kovarik et al. (2002b) and Kovarik et al., 2006)
						Sandimmune^®^ AUC_0–∞_ 74% ↑ (90% CI 49–104)			
					Rifampin (strong CYP3A inducer)	AUC 63% ↓ (90% CI 54–70)	Avoid coadministration of strong CYP3A inducers or increase the dose of everolimus to 10 or 20 mg QD	Clinical trial	(European Medicines Agency Committee for Medicinal Products For Human Use (CHMP); Kovarik et al. (2002a) and Food and Drug Administration (2008)
Imatinib (2001)	CYP3A	KIT, PDGFR, Bcr-Abl	40–60%	Dose proportional exposure	Ketoconazole (strong CYP3A inhibitor)	Single dose AUC_0–∞_ 40.1% ↑ (90% CI 31–49.9)	Be careful when combining imatinib with inhibitors of CYP3A	Clinical trial	Food and Drug Administration, (2008); European Medicines Agency Committee for Medicinal Products For Human Use (CHMP) (2006)
						N-desmethylimatinib AUC_0–∞_ 5% ↓ (90% CI −3–12.5)			
					Ritonavir (strong CYP3A inhibitor)	Steady-state AUC_0–24h_ 3.1% ↓ (90% CI −12.5–16.5)		Clinical trial	Van Erp et al. (2007)
						N-desmethylimatinib			
						AUC_0–24h_ 38.5% ↑ (90% CI 15.9–65.6)			
					Rifampin (strong CYP3A inducer)	AUC_0–∞_ 74% ↓ (90% CI 71–76)	Avoid coadministration of strong CYP3A inducers	Clinical trial	Bolton et al. (2004) and European Medicines Agency Committee for Medicinal Products For Human Use (CHMP) (2006)
						N-desmethylimatinib AUC_0–∞_ 11.7% ↓ (90% CI 3.3–19.4)			
Imatinib (2001)	CYP3A	KIT, PDGFR, Bcr-Abl	40–60%	Dose proportional exposure	Enzyme-inducing antiepileptic drugs (EIAEDs; e.g., carbamazepine, oxcarbazepine and phenytoin)	AUC_0–∞_ 72.5% ↓	Avoid coadministration of strong CYP3A inducers	Clinical trial	Wen et al. (2006)
					(mixed potency; carbamazepine and phenytoin are potent inducers; oxcarbazepine is a weak inducer)	N-desmethylimatinib AUC_0–∞_ 9.8% ↓			
					St John’s Wort (weak CYP3A inducer)	Study 1 AUC_0–∞_ 30.2% ↓ (90% CI 25–34.9)		Clinical trial	Frye et al. (2004)
						N-desmethylimatinib AUC_0–72h_ 4.1% ↑ (90% CI −8.4–18.3)			Smith et al. (2004)
						Study 2 AUC_0–∞_ 44% ↓ (90% CI 30–54)			
Lenvatinib (2015)	CYP3A	VEGFR	36–78%	Dose proportional exposure	Itraconazole (strong CYP3A inhibitor)	AUC_0–∞_ 14.5% ↑ (90% CI 8.5–20.9)	None	Clinical trial	Food and Drug Administration (2015b) and Shumaker et al. (2015)
					Rifampin (strong CYP3A inducer)	Single dose AUC_0–∞_ 30.6% ↑ (90% CI 22.7–39)		Clinical trial	Shumaker et al. (2014) and Food and Drug Administration (2015b)
						Multiple doses AUC_0–∞_ 18.2% ↓ (90% CI 8.7–26.7)			
Olaparib (2014)	CYP3A	PARP	38%	Dose-proportionality cannot be concluded based on available PK data	Itraconazole (strong CYP3A inhibitor)	Study 1; tablet AUC_0–∞_ 170% ↑ (90% CI 144–197) Study 2; capsule AUC 152% ↑ (95% CI 139–167)	Reduce dose of olaparib to 150 mg BID when combined with strong CYP3A inhibitors and reduce dose to 200 mg BID when combined with moderate CYP3A inhibitors (tablets)	Clinical trial	Food and Drug Administration (2014c) and Dirix et al. (2016)
								PBPK simulation	Pilla Reddy et al. (2019)
					Fluconazole (moderate CYP3A inhibitor)	Study 1; tablet AUC 126% ↑ (95% CI 115–130) Study 2; tablet AUC 121% ↑ (95% CI 114–128) Study 2; capsule AUC 98% ↑ (95% CI 92–105)		PBPK simulation	Food and Drug Administration (2014c)
								PBPK simulation	Pilla Reddy et al. (2019)
Olaparib (2014)	CYP3A	PARP	38%	Dose-proportionality cannot be concluded based on available PK data	Fluvoxamine (weak CYP3A inhibitor)	Tablet AUC 2% ↑ (95% CI 1–2)	Reduce dose of olaparib to 150 mg BID when combined with strong CYP3A inhibitors and reduce dose to 200 mg BID when combined with moderate CYP3A inhibitors (tablets)	PBPK simulation	Pilla Reddy et al. (2019)
						Capsule AUC 1% ↑ (95% CI 1–2)			
					Rifampin (strong CYP3A inducer)	Study 1; tablet AUC_0–∞_ 87% ↓ (90% CI 84–89)	Avoid coadministration of strong and moderate CYP3A inducers	Clinical trial	Food and Drug Administration, (2014c); Dirix et al. (2016)
						Study 2; capsule AUC 71% ↓ (95% CI 69–73)		PBPK simulation	Pilla Reddy et al. (2019)
					Efavirenz (moderate CYP3A inducer)	Study 1; tablet AUC 59% ↓ (95% CI 58–62)		PBPK simulation	Food and Drug Administration, (2014c)
						Study 2; tablet AUC 60% ↓ (95% CI 57–62)		PBPK simulation	Pilla Reddy et al. (2019)
						Study 2; capsule AUC 53% ↓ (95% CI 50–56)			
					Dexamethasone (weak CYP3A inducer)	Tablet AUC 0 (95% CI −1–0)		PBPK simulation	Pilla Reddy et al. (2019)
						Capsule AUC 0 (95% CI −1–0)			
Osimertinib (2015)	CYP3A	EGFR	37%	Dose proportional exposure	Itraconazole (strong CYP3A inhibitor)	AUC_0–∞_ 24.2% ↑ (90% CI 14.6–34.5)	None	Clinical trial	European Medicines Agency Committee for Medicinal Products For Human Use (CHMP) (2016a) and Vishwanathan et al. (2018)
						AZ5104 AUC_0–∞_ 8.3% ↑ (90% CI −5.6–24.2)			
						AZ7550 AUC 51% ↓ (90% CI 45–56.3)			
					Rifampin (strong CYP3A inducer)	AUC_0-24h_ 78.5% ↓ (90% CI 76.2–80.5)	Avoid coadministration of strong and moderate CYP3A inducers	Clinical trial	European Medicines Agency Committee for Medicinal Products For Human Use (CHMP) (2016a) and Vishwanathan et al. (2018)
						AZ5104 AUC_0-24h_ 81.2% ↓ (90% CI 78.8–83.4)			
						AZ7550 AUC_0-24h_ 29.8% ↑ (19.1–41.4)			
					Efavirenz (moderate CYP3A inducer)	AUC 42% ↓ (95% CI 40–44)		PBPK simulation	Reddy et al. (2018)
					Dexamethasone (weak CYP3A inducer)	AUC 0.001% ↓ (95% CI 0.001–0.001)		PBPK simulation	Reddy et al. (2018)
Palbociclib (2015)	CYP3A	CDK4/6	29%	Dose proportional exposure	Itraconazole (strong CYP3A inhibitor)	AUC_0–∞_ 86.8% ↑ (90% CI 72.9–101.9)	Avoid coadministration of strong CYP3A inhibitors or reduce dose of palbociclib to 75 mg QD	Clinical trial	Food and Drug Administration (2014d) and European Medicines Agency Committee for Medicinal Products For Human Use (CHMP) (2016b)
					Diltiazem (moderate CYP3A inhibitor)	AUC_0–216h_ 42% ↑		PBPK simulation	Yu et al. (2017)
					Verapamil (moderate CYP3A inhibitor)	AUC_0–216h_ 38% ↑		PBPK simulation	Yu et al. (2017)
					Fluvoxamine (weak inhibitor)	AUC_0–216h_ 0.4% ↑		PBPK simulation	Yu et al. (2017)
					Fluoxetine (weak CYP3A inhibitor)	AUC_0–216h_ 0.3% ↑		PBPK simulation	Yu et al. (2017)
					Rifampin (strong CYP3A inducer)	AUC_0–∞_ 85.2% ↓ (90% CI 81.4–88.2)	Avoid coadministration of strong CYP3A inducers	Clinical trial	Food and Drug Administration (2014d)
					Efavirenz (moderate CYP3A inducer)	AUC_0–168h_ 38% ↓		PBPK simulation	Yu et al. (2017)
Palbociclib (2015)	CYP3A	CDK4/6	29%	Dose proportional exposure	Modafinil (moderate CYP3A inducer)	AUC_0–∞_ 32% ↓	Avoid coadministration of strong CYP3A inducers	Clinical trial	European Medicines Agency Committee for Medicinal Products For Human Use (CHMP) (2016b)
Sonidegib (2015)	CYP3A	Smooth-ened	CL/F 67% V/F 213%	Dose proportional exposure with doses up to 400 mg, with higher dose less than pro-portional (due to dose-dependent absorption)	Ketoconazole (strong CYP3A inhibitor)	Study 1; healthy subjects AUC_0–240h_ 125% ↑ (90% CI 78–186)	Reduce dose of sonidegib to 200 mg every other day when combined with strong CYP3A inhibitors	Clinical trial	European Medicines Agency Committee for Medicinal Products For Human Use (CHMP), (2015c), and Food and Drug Administration (2015d)
						Study 2; cancer patients, sonidegib 1 day, ketoconazole 14 days AUC_0–24h_ 42% ↑ (90% CI 39–45)		PBPK simulation	Food and Drug Administration (2015d) and Einolf et al. (2017)
						Study 2; sonidegib 120 days, ketoconazole 120 days AUC_0-24h_ 253% ↑ (90% CI 231–276)			
						Study 2; sonidegib 133 days, ketoconazole 14 days AUC_0–24h_ 101% ↑ (90% CI 92–111)			
						Study 2; sonidegib QOD 133 days, ketoconazole 14 days AUC_0–24h_ 93% ↑ (90% CI 84–102)			
Sonidegib (2015)	CYP3A	Smooth-ened	CL/F 67% V/F 213%	Dose proportional exposure with doses up to 400 mg, with higher dose less than proportional (due to dose-dependent absorption)	Erythromycin (moderate CYP3A inhibitor)	Sonidegib 1 day, erythromycin 14 days AUC_0–24h_ 36% ↑ (90% CI 33–39)	Reduce dose of sonidegib to 200 mg every other day when combined with strong CYP3A inhibitors	PBPK simulation	Food and Drug Administration (2015d) and Einolf et al. (2017)
						Sonidegib 120 days, erythromycin 120 days AUC_0–24h_ 179% ↑ (90% CI 76–361)			
						Sonidegib 133 days, erythromycin 14 days AUC_0–24h_ 79% ↑ (90% CI 71–86)			
					Rifampin (strong CYP3A inducer)	Study 1; healthy subjects AUC_0–240h_ 72.4% ↓ (90% CI 65.1–78.1)	Avoid coadministration of strong CYP3A inducers, but if necessary, consider to increase the dose to 400–800 mg	Clinical trial	European Medicines Agency Committee for Medicinal Products For Human Use (CHMP) (2015c) and Food and Drug Administration (2015d)
						Study 2; cancer patients, sonidegib 1 day, rifampin 14 days AUC_0–24h_ 66% ↓ (90% CI 63–68)		PBPK simulation	Food and Drug Administration (2015d) and Einolf et al. (2017)
						Study 2; sonidegib 120 days, rifampin 120 days AUC_0-24h_ 88% ↓ (90% CI 87–89)			
						Study 2; sonidegib 133 days, rifampin 14 days AUC_0–24h_ 80% ↓ (90% CI 78–82)			
Sonidegib (2015)	CYP3A	Smooth-ened	CL/F 67% V/F 213%	Dose proportional exposure with doses up to 400 mg, with higher dose less than proportional (due to dose-dependent absorption)	Efavirenz (moderate CYP3A inducer)	Sonidegib 1 day, efavirenz 14 days AUC_0–24h_ 29% ↓ (90% CI 26–31)	Avoid coadministration of strong CYP3A inducers, but if necessary, consider to increase the dose to 400–800 mg	PBPK simulation	Food and Drug Administration (2015d) and Einolf et al. (2017)
						Sonidegib 120 days, efavirenz 120 days AUC_0-24h_ 65% ↓ (90% CI 62–67)			
						Sonidegib 133 days, efavirenz 14 days AUC_0–24h_ 53% ↓ (90% CI 50–56)			
Sunitinib (2006)	CYP3A	VEGFR	40%	Dose proportional exposure	Ketoconazole (strong CYP3A inhibitor)	Sum sunitinib and SU12662 AUC_0–∞_ 51% ↑	Reduce dose of sunitinib to 37,5 mg QD in GIST and MRCC patients and to 25 mg QD in pancreatic/NET patients when combined with strong CYP3A inhibitors	Clinical trial	Food and Drug Administration (2005)
					Grapefruit juice (moderate CYP3A inhibitor)	AUC_0–24h_ 11% ↑		Clinical trial	Van Erp et al. (2011)
					Rifampin (strong CYP3A inducer)	Sum sunitinib and SU12662 AUC_0–∞_ 46% ↓	Increase the dose of sunitinib in steps of 12.5 mg with a maximum of 87.5 mg QD when combined with CYP3A inducers	Clinical trial	Food and Drug Administration (2005)

### Drugs Without Active Metabolites

#### Ceritinib

When the strong CYP3A inhibitor ketoconazole was combined with a single-dose of ceritinib, the AUC_0–∞_ of ceritinib increased by 190% (*n* = 19) (Food and Drug Administration, 2014a). In a PBPK study the effect of ketoconazole on steady-state exposure of ceritinib was simulated. Steady-state exposure increased by 51% (Food and Drug Administration, 2014a). The difference between the effect of ketoconazole on single-dose and steady-state ceritinib concentrations can be explained by the auto-inhibition of CYP3A4 by ceritinib. Hereby, the fraction of ceritinib metabolized by CYP3A4 will be decreased at steady-state concentrations, thus the effect of a strong inhibitor will be smaller. (Food and Drug Administration, 2014a). The moderate inhibitor fluconazole increased the steady-state exposure of ceritinib by 37% in a PBPK simulation (Food and Drug Administration, 2014a). The strong CYP3A inducer rifampin decreased the AUC _0–∞_ of single-dose ceritinib by 70% (*n* = 19) and it was predicted to decrease the AUC on steady-state by 67%. In a simulation study, the moderate inducer efavirenz decreased the AUC of ceritinib by approximately half with 43% (Food and Drug Administration, 2014a).

#### Cobimetinib

**FIGURE 2:**
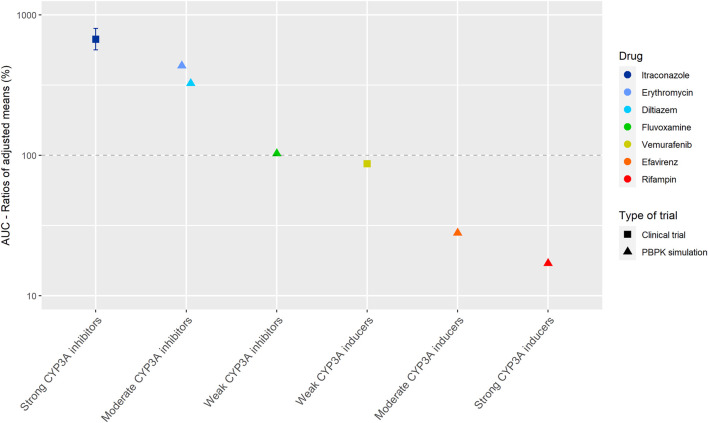
Overview of the results from DDI studies of cobimetinib combined with CYP3A inhibitors and inducers. The coloured symbols represent the increase or decrease in AUC caused by the interacting drug, expressed as adjusted mean ±90% confidence interval (if available). The dashed line represents the baseline AUC (Food and Drug Administration, 2014b).

Figure 2 shows the results of the DDI studies conducted with cobimetinib. It can be seen that CYP3A based DDIs have a large influence on the exposure to cobimetinib. The strong inhibitor itraconazole increased the AUC_0–∞_ of cobimetinib by almost 600% (*n* = 15) (Food and Drug Administration, 2014b). The moderate CYP3A inhibitors erythromycin and diltiazem increased the AUC by around 300% in a PBPK simulation, which is half the effect of strong inhibitors, while weak inhibitors had no effect (Food and Drug Administration, 2014b; Budha et al., 2016). The effect of rifampin on the exposure of cobimetinib was studied in a PBPK simulation study instead of a clinical trial, which is in contrast with most DDI studies performed with rifampin. In this simulation the AUC of cobimetinib decreased by 83% when combined with rifampin (Food and Drug Administration, 2014b). Furthermore, the effect of the moderate CYP3A inducer efavirenz was studied in a PBPK simulation and a decrease in AUC of 72% was predicted (Food and Drug Administration, 2014b; Budha et al., 2016). The weak inducer vemurafenib showed a decrease in AUC_0–24h_ of only 13% in a clinical trial (*n* = unknown) (Food and Drug Administration, 2014b).

#### Everolimus

The strong inhibitor ketoconazole increased the AUC_0–∞_ of everolimus by 1,430% (*n* = 12) (Kovarik et al., 2005b; Food and Drug Administration, 2008). Therefore, it is not recommended to coadminister strong CYP3A4 inhibitors with everolimus (Food and Drug Administration, 2008). The effect size of moderate inhibitors was around 25% compared with ketoconazole (increase in exposure of 340% for erythromycin (*n* = 16), 250% for verapamil (*n* = 16), 270% for imatinib (*n* = unknown), and 121% as average for two different cyclosporin formulations (*n* = 12) (European Medicines Agency Committee for Medicinal Products For Human Use (CHMP); Kovarik et al., 2002a; Kovarik et al., 2002b; Kovarik et al., 2005a; Kovarik et al., 2005c; Kovarik et al., 2006). Rifampin decreased the AUC of everolimus by 63% (*n* = 12) (Kovarik et al., 2002a). The effect of the moderate inhibitors was small compared with the strong inhibitor ketoconazole. An explanation for this finding is that ketoconazole also inhibits P-glycoprotein (P-gp), which influences the pharmacokinetics (PK) of everolimus in addition to CYP3A (European Medicines Agency Committee for Medicinal Products For Human Use (CHMP), 2014; Ravaud et al., 2014).

#### Lenvatinib

Lenvatinib is for more than 80% metabolized by CYP3A to different metabolites *in vitro*. Furthermore, *in vitro* data suggests that lenvatinib is a substrate for P-gp. But *in vivo*, oxidation by aldehyde oxidase and glutathione conjugation play an important role in the metabolism of lenvatinib, next to the metabolism *via* CYP3A (Food and Drug Administration, 2015b). Since the potency of lenvatinib is at least 20 times higher than of the metabolites, the metabolites were considered inactive (Shumaker et al., 2014; Food and Drug Administration, 2015b). The strong CYP3A inhibitor ketoconazole increased the AUC_0–∞_ of lenvatinib by 15% (*n* = 18) (Food and Drug Administration, 2015b; Shumaker et al., 2015). The strong CYP3A inducer rifampin decreased the AUC_0–∞_ of lenvatinib by 18% when multiple doses were given (*n* = 15) (Shumaker et al., 2014; Food and Drug Administration, 2015b). In contrast, a single dose of rifampin increased the AUC_0–∞_ of lenvatinib by 31%. Shumaker et al. explained this by a presystemic inhibition of P-gp, which is consistent with the study of Rietman et al. who described that rifampin can inhibit the efflux of drugs into the intestinal lumen (Reitman et al., 2011; Shumaker et al., 2014). The marginal effects of ketoconazole and rifampin on the lenvatinib AUC suggest that the role of CYP3A in the metabolism of lenvatinib is small. In addition, the effects of ketoconazole and rifampin on the AUC of lenvatinib could also be caused by inhibition and induction of P-gp, because both ketoconazole and rifampin have an effect on P-gp (European Medicines Agency Committee for Medicinal Products For Human Use (CHMP), 2014b; Food and Drug Administration, 2015b).

#### Olaparib

Clinical DDI studies investigated the influence of itraconazole and rifampin on the AUC of olaparib administered as tablets (Food and Drug Administration, 2014c; Dirix et al., 2016). In PBPK simulations, the effect of inhibitors and inducers on the AUC of olaparib formulated as capsules was simulated. The effect on olaparib tablets and capsules were predicted to be similar (Pilla Reddy et al., 2019).

The strong CYP3A inhibitor itraconazole increased the AUC_0–∞_ of olaparib by 170% (*n* = 59) (Food and Drug Administration, 2014c; Dirix et al., 2016). The moderate inhibitor fluconazole increased the AUC of olaparib with an average of 115% in three PBPK simulations (Food and Drug Administration, 2014c; Pilla Reddy et al., 2019). Furthermore, the weak inhibitor fluvoxamine, was simulated to have no effect on the AUC of olaparib (Pilla Reddy et al., 2019). Rifampin, a strong CYP3A inducer, decreased the olaparib AUC _0–∞_ by 87% (*n* = 22) (Food and Drug Administration, 2014c). The moderate inducer efavirenz decreased the AUC of olaparib by approximately 75%, compared with rifampin, with a decrease of 60% in a PBPK simulation (Pilla Reddy et al., 2019). The weak inducer dexamethasone, was simulated to have no effect on the AUC of olaparib (Pilla Reddy et al., 2019).

#### Palbociclib

**FIGURE 3:**
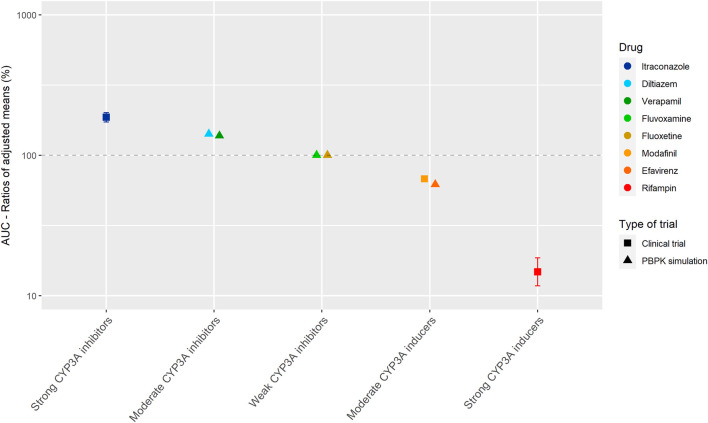
Overview of the results from DDI studies of palbociclib combined with CYP3A inhibitors and inducers. The coloured symbols represent the increase or decrease in AUC caused by the interacting drug, expressed as adjusted mean ±90% confidence interval (if available). The dashed line represents the baseline AUC (Food and Drug Administration, 2014d; European Medicines Agency Committee for Medicinal Products For Human Use (CHMP), 2016b; Yu et al., 2017).

Figure 3 shows the results of the DDI studies performed with palbociclib. The strong inhibitor itraconazole increased the palbociclib AUC_0–∞_ by 87% (*n* = 12) (Food and Drug Administration, 2014d; European Medicines Agency Committee for Medicinal Products For Human Use (CHMP), 2015b). The moderate CYP3A inhibitors diltiazem and verapamil were simulated to increase the AUC_0–216h_ of palbociclib by half compared with itraconazole, with an increase of 40% (Food and Drug Administration, 2014d; Yu et al., 2017). No effect of the weak inhibitors fluvoxamine and fluoxetine on the AUC_0–216h_ of palbociclib was predicted in a simulation study (Yu et al., 2017). Moderate CYP3A inducers decreased the palbociclib AUC by approximately half compared with strong CYP3A inducers. The strong inducer rifampin decreased the AUC_0–∞_ of palbociclib by 85% (*n* = 14) (Food and Drug Administration, 2014d). The moderate inducer efavirenz decreased the AUC_0–168h_ by 38% in a simulation study and modafinil decreased the AUC_0–∞_ by 32% in a clinical trial (*n* = 14) (European Medicines Agency Committee for Medicinal Products For Human Use (CHMP), 2016b; Yu et al., 2017).

#### Sonidegib

In a clinical trial with healthy subjects, the strong CYP3A inhibitor ketoconazole increased the AUC_0–240h_ of sonidegib 800 mg by 125% (same was simulated for sonidegib 200 mg) (parallel study; *n* = 16 in control group and *n* = 15 in combination group) (European Medicines Agency Committee for Medicinal Products For Human Use (CHMP), 2015c; Food and Drug Administration, 2015d; Einolf et al., 2017). Ketoconazole was simulated to increase the AUC_0–24h_ of sonidegib given as a single dose by 42% in cancer patients (Food and Drug Administration, 2015d; Einolf et al., 2017). The smaller effect of ketoconazole in cancer patients, can be explained by a decreased hepatic clearance with an elimination half-life of 28 days in cancer patients, and 10 days in healthy subjects (Food and Drug Administration, 2015d). After long-term exposure to sonidegib, ketoconazole was simulated to increase the AUC_0–24h_ by 101–253%, dependent on the duration of combined use (Food and Drug Administration, 2015d; Einolf et al., 2017).

The moderate CYP3A inhibitor erythromycin increased the AUC_0–24h_ of sonidegib given as a single dose by 36% (Food and Drug Administration, 2015d; Einolf et al., 2017). The AUC_0–24h_ of sonidegib given long-term was increased by 79–179%, dependent on the duration of combined use with erythromycin (Food and Drug Administration, 2015d; Einolf et al., 2017). Compared with the simulations for ketoconazole, according to the same treatment schedule, the increases in sonidegib AUC were more than half.

In a clinical trial with healthy subjects, the strong CYP3A inducer rifampin decreased the AUC_0–240h_ of sonidegib 800 mg by 72.4% (same was simulated for sonidegib 200 mg) (parallel study; *n* = 16 in control group and *n* = 16 in combination group) (European Medicines Agency Committee for Medicinal Products For Human Use (CHMP), 2015c; Food and Drug Administration, 2015d; Einolf et al., 2017). Rifampin was simulated to decrease the AUC_0–24h_ of sonidegib given as a single dose by 66% in cancer patients (Food and Drug Administration, 2015d; Einolf et al., 2017). The smaller decrease in cancer patients can be explained by a decreased hepatic clearance. The AUC_0–24h_ of sonidegib was decreased by 80–88% when sonidegib given long-term and rifampin were combined, dependent on the duration of combined use (Food and Drug Administration, 2015d; Einolf et al., 2017).

The moderate CYP3A inducer efavirenz was simulated to decrease the AUC_0–24h_ of sonidegib given as a single dose by 29% (Food and Drug Administration, 2015d; Einolf et al., 2017). Efavirenz decreased the AUC_0–24h_ of sonidegib given long-term by 53–65%, dependent on the duration of combined use (Food and Drug Administration, 2015d; Einolf et al., 2017). Compared with the simulations of rifampin, according to the same treatment schedule, a decrease of approximately 70% was seen in sonidegib steady-state AUC.

To summarize, the interacting effect on sonidegib is influenced by the patient population and duration of therapy with sonidegib and the interacting agent.

### Drugs With Active Metabolites

#### Alectinib

Alectinib is mainly metabolized by CYP3A to the active metabolite M4. Alectinib and M4 show a similar potency and plasma protein binding *in vitro* (Fowler et al., 2017; Morcos et al., 2017). Therefore, the sum of alectinib and M4 concentration was reported as the pharmacologically active exposure in the DDI studies with posaconazole and rifampin (Morcos et al., 2017).

**FIGURE 4:**
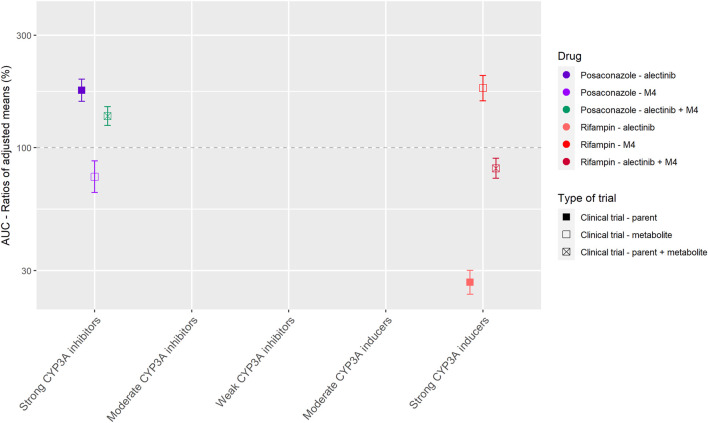
Overview of the results from DDI studies of alectinib combined with CYP3A inhibitors and inducers. The coloured symbols represent the increase or decrease in AUC caused by the interacting drug, expressed as adjusted mean ±90% confidence interval (if available). The dashed line represents the baseline AUC (Food and Drug Administration, 2015a; Morcos et al., 2017).

Figure 4 shows the results of the DDI studies performed with alectinib. The strong inhibitor posaconazole increased the exposure to the sum of alectinib and M4 by 36% (*n* = 17) (Food and Drug Administration, 2015a; Morcos et al., 2017). The strong inducer rifampin decreased the sum of exposure by 18% (*n* = 24) (Food and Drug Administration, 2015a; Morcos et al., 2017). Based on the small effects of posaconazole and rifampin, the effects of other CYP3A inhibitors and inducers on the exposure of alectinib and M4 were considered clinically irrelevant.

#### Dabrafenib

Dabrafenib is partially metabolized to active metabolites. It is firstly oxidized by CYP enzymes to hydroxy-dabrafenib, which is further oxidized to carboxy-dabrafenib. Carboxy-dabrafenib is converted to desmethyl-dabrafenib *via* a non-enzymatic process or excreted in urine or bile. Subsequently, desmethyl-dabrafenib is oxidized to other metabolites (Bershas et al., 2013). Dabrafenib auto-induces its metabolism *via* CYP3A4 (Food and Drug Administration, 2012). Hydroxy-dabrafenib and desmethyl-dabrafenib show a similar potency and may contribute to the clinical activity of dabrafenib, on the other hand carboxy-dabrafenib does not relevantly contribute to the activity (Suttle et al., 2015).

The strong inhibitor ketoconazole increased the AUC_0–12h_ of dabrafenib and the metabolites hydroxy-dabrafenib and desmethyl-dabrafenib by 71, 82, and 68%, respectively, while the AUC_0–12h_ of carboxy-dabrafenib decreased by 16% (*n* = 16) (Suttle et al., 2015; European Medicines Agency Committee for Medicinal Products For Human Use (CHMP), 2018). In the DDI study with the strong inducer rifampin the opposite was seen, the AUCs of dabrafenib and desmethyl-dabrafenib decreased by 34 and 30%, respectively, and the AUC of the inactive carboxy-dabrafenib increased by 73% (*n* = 23) (European Medicines Agency Committee for Medicinal Products For Human Use (CHMP), 2018). These results for both parent and metabolites when combined with a strong inhibitor versus a strong inducer were as expected because the conversion of dabrafenib, hydroxy-dabrafenib, and desmethyl-dabrafenib is mediated by CYP enzymes and thus influenced by inhibitors and inducers of CYP3A. On the contrary, the non-enzymatic conversion of carboxy-dabrafenib is not affected by CYP3A inhibitors and inducers (Bershas et al., 2013). The comparable or even higher increase in AUC for hydroxy-dabrafenib and desmethyl-dabrafenib compared to the parent, indicates higher involvement of CYP3A in elimination of the metabolites compared to their production (Suttle et al., 2015; European Medicines Agency Committee for Medicinal Products For Human Use (CHMP), 2018).

#### Imatinib

Imatinib is mainly metabolized by CYP3A. Other CYP enzymes play a minor role. Auto-inhibition of CYP3A by imatinib was shown *in vitro*, but no *in vivo* data is available (Food and Drug Administration, 2001). The main metabolite is N-desmethylimatinib also known as CGP 74588. N-desmethylimatinib is as potent as the parent compound *in vitro*. The exposure to N-desmethylimatinib is approximately 10% compared to the exposure to imatinib, therefore the effect of the metabolite is considered clinically irrelevant (Peng et al., 2005; Whirl-Carrillo et al., 2012).

Ketoconazole in combination with a single dose of imatinib increased the imatinib AUC_0–∞_ by 40% (*n* = 14) (Food and Drug Administration, 2001; European Medicines Agency Committee for Medicinal Products For Human Use (CHMP), 2006). Ritonavir combined with imatinib, at imatinib steady-state, decreased the imatinib AUC_0–24h_ by 3% (*n* = 11) (Van Erp et al., 2007). According to the Flockhart Table, ritonavir and ketoconazole share the same interaction potential (Flockhart, 2007). But ritonavir is also an inhibitor of CYP2D6 and inducer of CYP2C19 (Flockhart, 2007), which both play a minor role in the metabolism of imatinib (European Medicines Agency Committee for Medicinal Products For Human Use (CHMP), 2006; Whirl-Carrillo et al., 2012). Especially the induction of CYP2C19 could be an explanation for the difference seen between the effects of ketoconazole and ritonavir. Furthermore, the difference could be caused by a shift to alternative elimination routes when imatinib is administered chronically, especially because auto-inhibition of CYP3A was shown *in vitro* (Food and Drug Administration, 2001). The two described hypotheses are supported by the *in vitro* experiment of Van Erp et al. which showed that ritonavir completely inhibited the metabolism of imatinib *via* CYP3A, but in human liver microsomes by only 50% (Van Erp et al., 2007). In DDI studies with CYP3A inducers large effects of the drugs rifampin and enzyme-inducing antiepileptic drugs (EIAEDs) such as carbamazepine, oxcarbazepine and phenytoin on imatinib AUC were seen. The strong inducer rifampin decreased the AUC_0–∞_ of imatinib by 74% (*n* = 14) (Bolton et al., 2004; European Medicines Agency Committee for Medicinal Products For Human Use (CHMP), 2006). EIAEDs (mixed potency; carbamazepine and phenytoin are potent inducers, oxcarbazepine is a weak inducer (Riva et al., 1996)) decreased the AUC_0–∞_ of imatinib by 72.5% (*n* = 50; *n* = 27 in EIAED group and *n* = 23 in non-EIAED group) (Wen et al., 2006). The effect of St John’s Wort on imatinib exposure was smaller with an average decrease of 37% in 2 studies (*n* = 12 in study Frye et al.; *n* = 10 in study Smith et al.) (Frye et al., 2004; Smith et al., 2004). To summarize, DDI studies with mostly strong CYP3A inhibitors and inducers were performed. The effects of these drugs on imatinib were variable. This can be due to differences in study design, characteristics of the interacting drugs and also the inter-individual variability of 40–60% will have an effect (Food and Drug Administration, 2001).

#### Osimertinib

Osimertinib is converted into different metabolites by predominantly CYP3A, among which the active metabolites AZ5104 and AZ7550. The exposure to the active metabolites is, however, less than 10% of the total drug exposure, therefore the effects of the metabolites are considered clinically irrelevant (Vishwanathan et al., 2018). Next to the metabolism by CYP3A, in *in vitro* studies CYP1A2, CYP2A6, CYP2C9, CYP2E1 also play a minor role in the metabolism of osimertinib (Dickinson et al., 2016; Vishwanathan et al., 2018). *In vitro* studies also showed that osimertinib is an inhibitor of CYP3A, but no *in vivo* data is available (Food and Drug Administration, 2015c).

The strong inhibitor itraconazole increased the AUC_0–∞_ of osimertinib by 24% (*n* = 38) (European Medicines Agency Committee for Medicinal Products For Human Use (CHMP), 2015a; Vishwanathan et al., 2018). On the other hand, the effect of rifampin on osimertinib exposure was large, rifampin decreased the AUC_0–24h_ by 78.5% (*n* = 32) (European Medicines Agency Committee for Medicinal Products For Human Use (CHMP), 2016a; Vishwanathan et al., 2018). The moderate inducer efavirenz was simulated to decrease the exposure by approximately 50% compared with rifampin, with a decrease in AUC of 42% (Reddy et al., 2018). Dexamethasone, a weak CYP3A inducer, had no effect on the AUC of osimertinib in a PBPK simulation (Reddy et al., 2018).

The presence of a clinically relevant effect for the interaction of osimertinib with rifampin, while it was lacking for the interaction between osimertinib and itraconazole, could be explained by the fact that rifampin induces multiple enzymes and transporters, and that, next to CYP3A, other CYP enzymes play a role in the metabolism of osimertinib (Vishwanathan et al., 2018). For the drugs tivozanib and ixazomib, also a clinically relevant effect was shown for rifampin, while it was lacking for a CYP3A inhibitor (Cotreau et al., 2015; Gupta et al., 2018; Vishwanathan et al., 2018).

#### Sunitinib

Sunitinib is metabolized by CYP3A to the active metabolite SU12662, which is equally potent (Food and Drug Administration, 2005). SU12662 is metabolized further by CYP3A and transported by P-gp (Heath et al., 2011).

The strong inhibitor ketoconazole increased the sum of the AUC_0–∞_ of sunitinib and SU12662 only by 51% (*n* = 27) (Food and Drug Administration, 2005). Grapefruit juice, a moderate CYP3A inhibitor, increased the AUC _0–24h_ of sunitinib by 11%, which was considered negligible (*n* = 8) (Van Erp et al., 2011). In this study only the AUC of sunitinib was measured and not the AUC of the metabolite SU12662. Grapefruit juice mainly inhibits intestinal CYP3A with little effect on hepatic CYP3A, while ketoconazole inhibits both (Saito et al., 2005). In addition, the small increase in AUC could be explained by the fact that in the study with ketoconazole (Food and Drug Administration, 2005), only a single dose of sunitinib was administered in contrast to the multiple dosing in the grapefruit juice study (Van Erp et al., 2011), which could lead to a shift to other metabolic pathways. The strong CYP3A inducer rifampin reduced the sum of the AUC_0–∞_ of sunitinib and SU12662 by 46% (*n* = 28) (Food and Drug Administration, 2005).

## Discussion

Most currently used oral targeted anticancer drugs have a narrow therapeutic range. Furthermore, most of these drugs are substrates of CYP3A and are, therefore, prone to DDIs with inhibitors or inducers of CYP3A. It is of crucial importance for clinical practice to have guidelines on how to deal with these DDIs in cases where data is lacking, which might be the case early after drug approval. This study reviewed the literature for DDI studies performed with twelve oral anticancer drugs. Based on this data, we formulated recommendations for clinical practice on how to deal with DDIs of oral anticancer drugs when only data from strong inducers or inhibitors is available.

**FIGURE 5:**
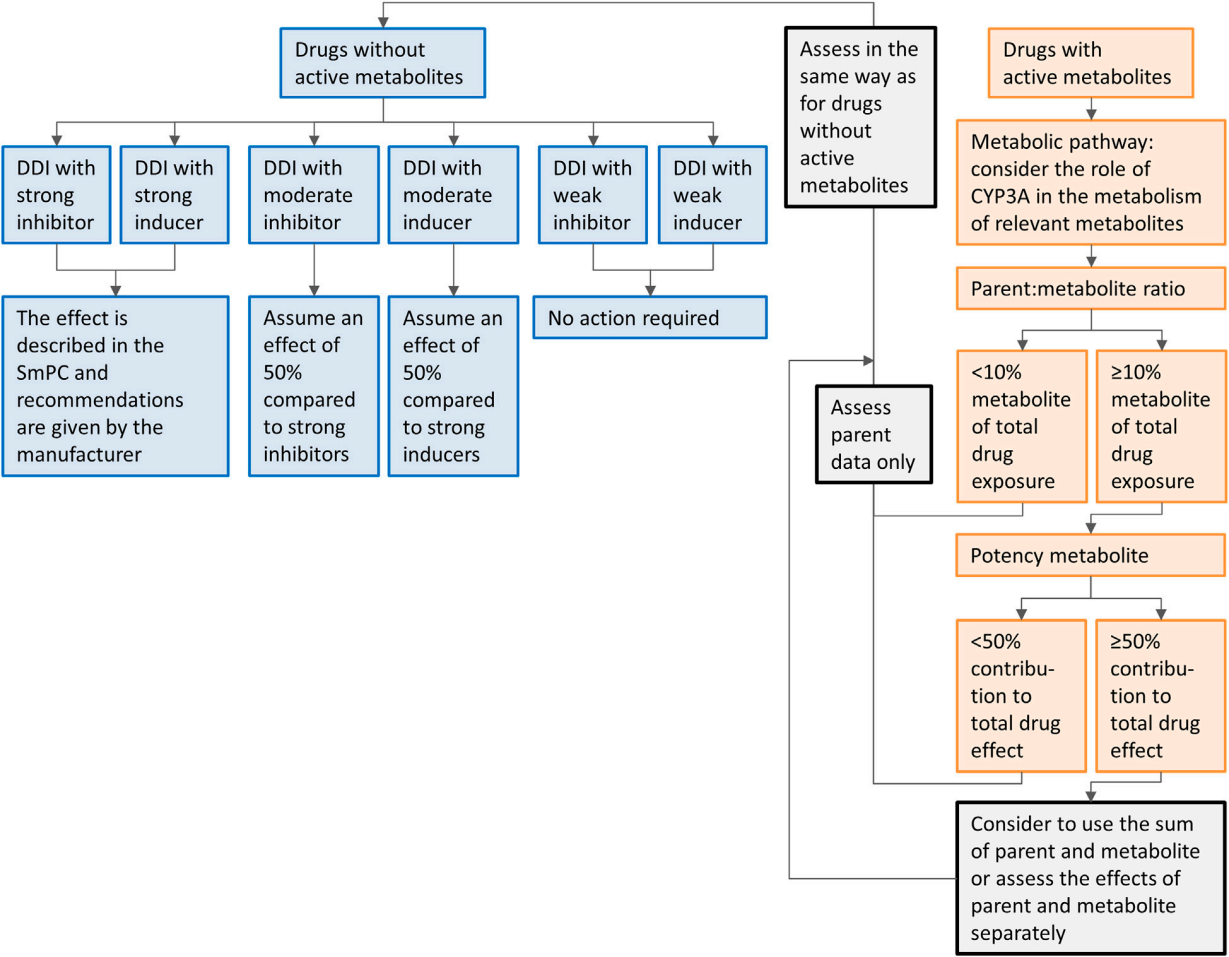
Flowchart of the recommendations on how to handle DDIs for oral anticancer drugs metabolized by CYP3A if only clinical data from strong CYP3A inhibitors or inducers is available. Caution should be taken while using the flowchart for drugs in which auto-induction or -inhibition plays a role and drug-drug interaction studies are not performed on steady-state, or for drugs with nonlinear dose-exposure relationships.

In our approach, we extrapolated results from dedicated DDI studies with strong inhibitors and inducers to clinical practice. Since the extrapolation of the effects of CYP3A inhibitors and inducers is more complex in the presence of active metabolites, separate recommendations are given for the drugs metabolized to inactive and with active metabolites. The recommendations are summarized in a flowchart (Figure 5). When interested in a victim drug without active metabolites, start in the left of the figure in the upper blue box. Follow the flowchart depending on the characteristics (inhibitor or inducer; interaction potential) of the drug you are interested in. The last box will show you our recommendation regarding the interaction. When interested in a victim drug with active metabolites, start in the right of the figure in the upper orange box. When the metabolite contributes less than 10% to total drug exposure or less than 50% to total drug effect, the presence of an active metabolite can be neglected. Therefore, the part of the flowchart for drugs without active metabolites can be followed. If the metabolite has a relevant contribution to total drug exposure and effect, the part of the flowchart for drugs without active metabolites can be followed, using the sum of parent and metabolite, or assessing the effect of parent and metabolite separately.

For the studied drugs without active metabolites, Tables 1 and 2 show that the effect of moderate CYP3A inhibitors on the AUC is roughly approximate to 50% of the effect of the strong inhibitors. The same effect can be seen for moderate inducers in comparison with strong inducers. Furthermore, it can be noted that weak inhibitors and inducers had marginal effects on the exposure of the studied drugs. In Figures 2 and 3, these results are visualized for the drugs cobimetinib and palbociclib, which gives a good representation of the effects seen for all seven drugs without active metabolites (the Supplementary Material shows figures for the other drugs).

Regarding drug selection for this review we made the following decisions. Drugs which have been approved for solid tumors from January 1st, 2013 to December 31st, 2015, and three drugs (everolimus, imatinib, sunitinib) authorized before 2013 based on the availability of relevant clinical data were selected. This resulted in a selection of twelve drugs. This was decided since 1) no difference is to be expected in quality of PBPK simulations performed in early years (2013–2015) compared to later years, and 2) the results of all twelve analyzed drugs in this review roughly indicate the same direction on the extrapolation of the effects of DDI studies. For the twelve drugs selected in our analysis, only for sunitinib and palbociclib a clinical trial was performed with a moderate CYP3A inhibitor and inducer, respectively. Also for the seven drugs that were approved after 2015 and met the inclusion criteria regarding metabolism and indication (abemaciclib, brigatinib, entrectinib, larotrectinib, lorlatinib, neratinib, and ribociclib) no clinical DDI studies with moderate inhibitors/inducers, but only PBPK simulations were performed (or no DDI studies at all). Furthermore, we decided to focus on oral anticancer drugs in our review. However, our recommendations are probably also applicable to other drugs metabolized by CYP3A.

It is important to take into account the following, regarding our recommendations. First, a large variability in the PK after multiple doses occurred in the studied drugs, with a range of 23–78%. Similarly, Verheijen et al. showed that there is a high inter-individual variability in the exposure to kinase inhibitors (Verheijen et al., 2017). This is also reflected by the large variability in the effect of CYP3A inhibitors and inducers for some drugs. Possibly, this variability in exposure could partly be explained by the highly variable CYP3A4 activity among patients, which is for 60–90% genetically determined (Özdemir et al., 2000; Westlind-Johnsson et al., 2003). For example, the CYP3A4*22 polymorphism has been described, resulting in a two-fold increase of the formation of a non-functional variant of CYP3A4 (Wang et al., 2011). If the CYP3A4 activity is decreased by a genetic polymorphism, the magnitude of the effect of a CYP3A inhibitor will theoretically be decreased. Furthermore, caution should be taken while using the flowchart for drugs in which auto-induction or -inhibition plays a role and drug-drug interaction studies are not performed on steady-state, or for drugs with nonlinear dose-exposure relationships. In these cases it might not be possible to extrapolate results from DDI studies with strong inhibitors and inducers, or dose recommendations based on these results. While interpreting the results of this review it is necessary to bear in mind this large variability in PK, and the exceptions in which our recommendations might not be applicable.

Next to the results of the drugs without active metabolites, Tables 1 and 2 show that for drugs that have active metabolites the results are less straightforward. As a visual example Figure 4 was made, which shows the effect of interacting drugs on the AUC of the parent drug alectinib and its active metabolite (similar figures are presented in the Supplementary Material for the other studied drugs). There are three factors to take into account while interpreting the results of DDI studies with drugs with active metabolites. Firstly, the metabolic pathway is important. For example, in case of dabrafenib not only the parent, but also two of the active metabolites are metabolized by CYP3A, whereas the third metabolite is converted non-enzymatically. This results in an effect of CYP3A inhibitors and inducers on both parent and some of the metabolites, but not all of them. Secondly, the ratio between parent and metabolites should be taken into account. As a cut-off value a contribution of less than 10% of the metabolite to total drug exposure could be used. This is in line with the EMA recommendation to characterize metabolites structurally that contribute to more than 10% of the AUC of a drug in in vitro studies (European Medicines Agency Committee for Medicinal Products For Human Use (CHMP), 2015b). An example of a drug with an active metabolite which contributes to less than 10% of total drug exposure is osimertinib. Thirdly, the potency of the metabolites plays an important role. A cut-off value of 50% contribution to the total drug effect can be used when considering the relevance of the contribution of an active metabolite. This cut-off value is supported by the EMA (European Medicines Agency Committee for Medicinal Products For Human Use (CHMP), 2015b). Shown by the recommendation to conduct an *in vivo* DDI study not only for drugs where enzymes contribute to at least 25% of the overall elimination but also for drugs with pharmacologically active metabolites which contribute to 50% or more of the effect of the drug (and enzymes are involved in the formation or elimination of these metabolites) (European Medicines Agency Committee for Medicinal Products For Human Use (CHMP), 2015b). For example, if a metabolite is as potent as the parent drug, the effect of an interacting drug on the sum of parent and metabolite might be reported as measure of total drug activity, as was done in the case of alectinib and sunitinib.

A practical example for the drug palbociclib is given. The assumption of an effect of 50% in comparison to that of strong inhibitors and inducers can be used to extrapolate the advice of the manufacturer. In case of palbociclib, the standard dose is 125 mg once daily (QD). The manufacturer recommends to reduce the dose of palbociclib to 75 mg (QD) if combination with a strong CYP3A inhibitor cannot be avoided. In combination with a moderate CYP3A inducer it could be considered to reduce the dose with 50% compared with the reduction in combination with strong inhibitors. This would result in a dose of 100 mg QD (Food and Drug Administration, 2014d). A reason to reduce the dose of palbociclib is that a higher palbociclib exposure is associated with increased toxicity, specifically a larger decrease in absolute neutrophil count when compared with baseline. However, the limited data available on exposure-response and exposure-toxicity relationships could be a consideration to start with the standard starting dose and decrease the dose in case toxicity occurs (Flaherty et al., 2012; Food and Drug Administration, 2014d; Verheijen et al., 2017).

After initiation of therapy with oral anticancer drugs in a reduced or increased dose, attainment of adequate drug exposure could be monitored by means of Therapeutic Drug Monitoring. Many of the oral anticancer drugs show an exposure-efficacy and an exposure-toxicity relationship, the strength of the evidence for these relationships is and recommendations for target plasma trough levels are discussed by Verheijen et al. (Verheijen et al., 2017).

## Conclusion

In conclusion, DDIs are often very complex and dependent on multiple factors. But, if only data from strong CYP3A inhibitors or inducers is available, in case of drugs without active metabolites, a change in exposure of 50% for moderate inhibitors/inducers compared with strong inhibitors/inducers can be assumed. We therefore recommend to start with a 50% dose reduction compared with the advised reduction in combination with strong inhibitors, and with a 50% dose increase compared to the advised increase in combination with strong and inducers.

Since an effect of weak CYP3A inhibitors on the AUC of oral anticancer drugs is small in the twelve reviewed drugs, *a priori* dose adaptations are not indicated.

In the presence of active metabolites, the response on DDIs should be based on the metabolic pathway, the exposure to the metabolites compared with the parent drug and to the potency of the metabolites. Options are to ignore the presence of a metabolite (for example when a metabolite is not pharmacologically active or contributes minimal to the exposure of the drug) or to use the sum of the parent and metabolite (at least do this when parent and metabolite are equally potent).
