# Supplementary material for: A Review of CYP3A Drug-Drug Interaction Studies: Practical Guidelines for Patients Using Targeted Oral Anticancer Drugs
Source: Front Pharmacol. 2021 Aug 30;12:670862. doi: 10.3389/fphar.2021.670862 (PMC8435708; doi:10.3389/fphar.2021.670862)
Supplement: Supplementary file 1 [file DataSheet1.docx]

# Supplemental information *Figures for drugs without active metabolites*


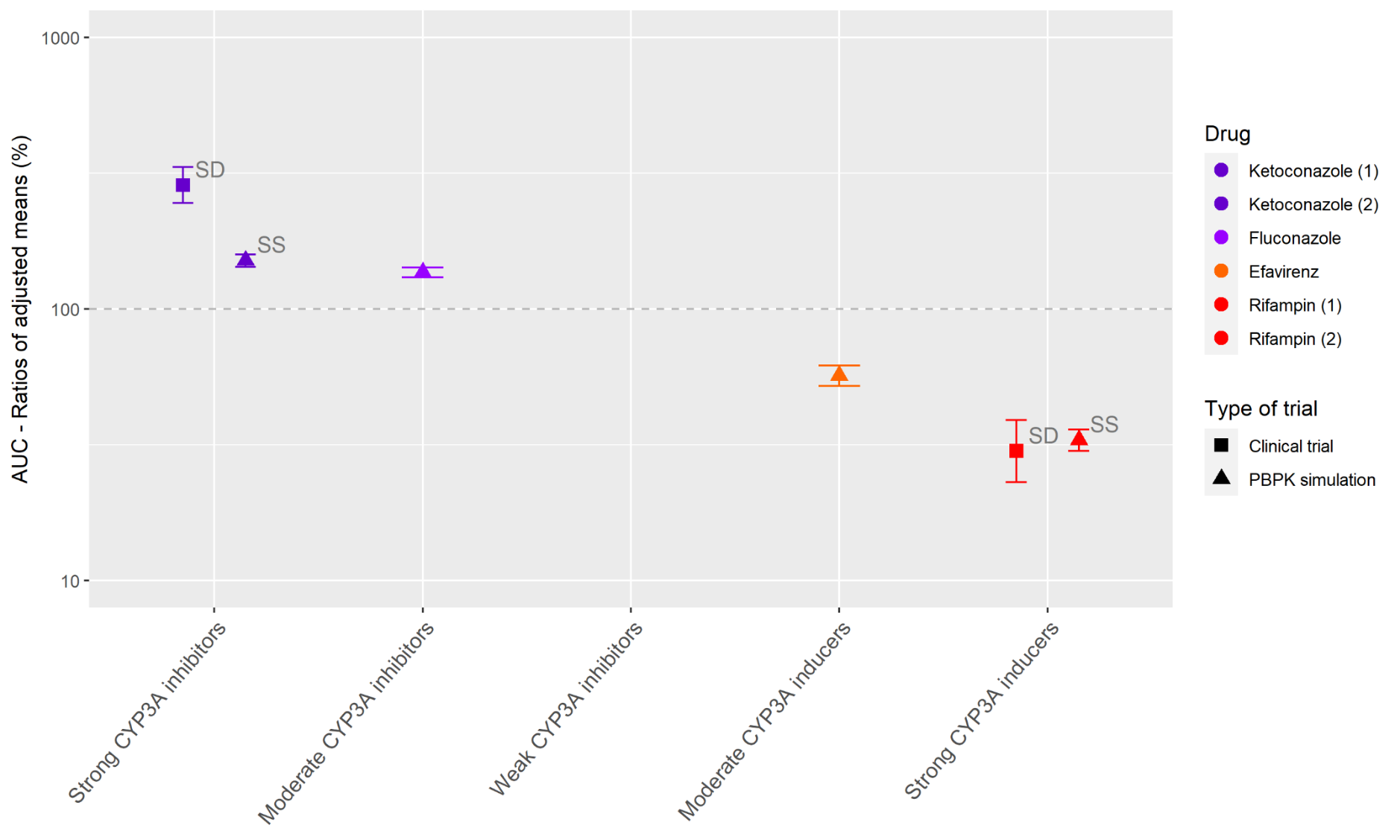

Supplemental Figure S1. Overview of the results from DDI studies of ceritinib combined with CYP3A inhibitors and inducers. The colored symbols represent the increase or decrease in AUC caused by the interacting drug, expressed as adjusted mean ± 90% confidence interval (if available). The dashed line represents the baseline AUC. SD = single dose; SS = steady-state (Food and Drug Administration, 2014a).


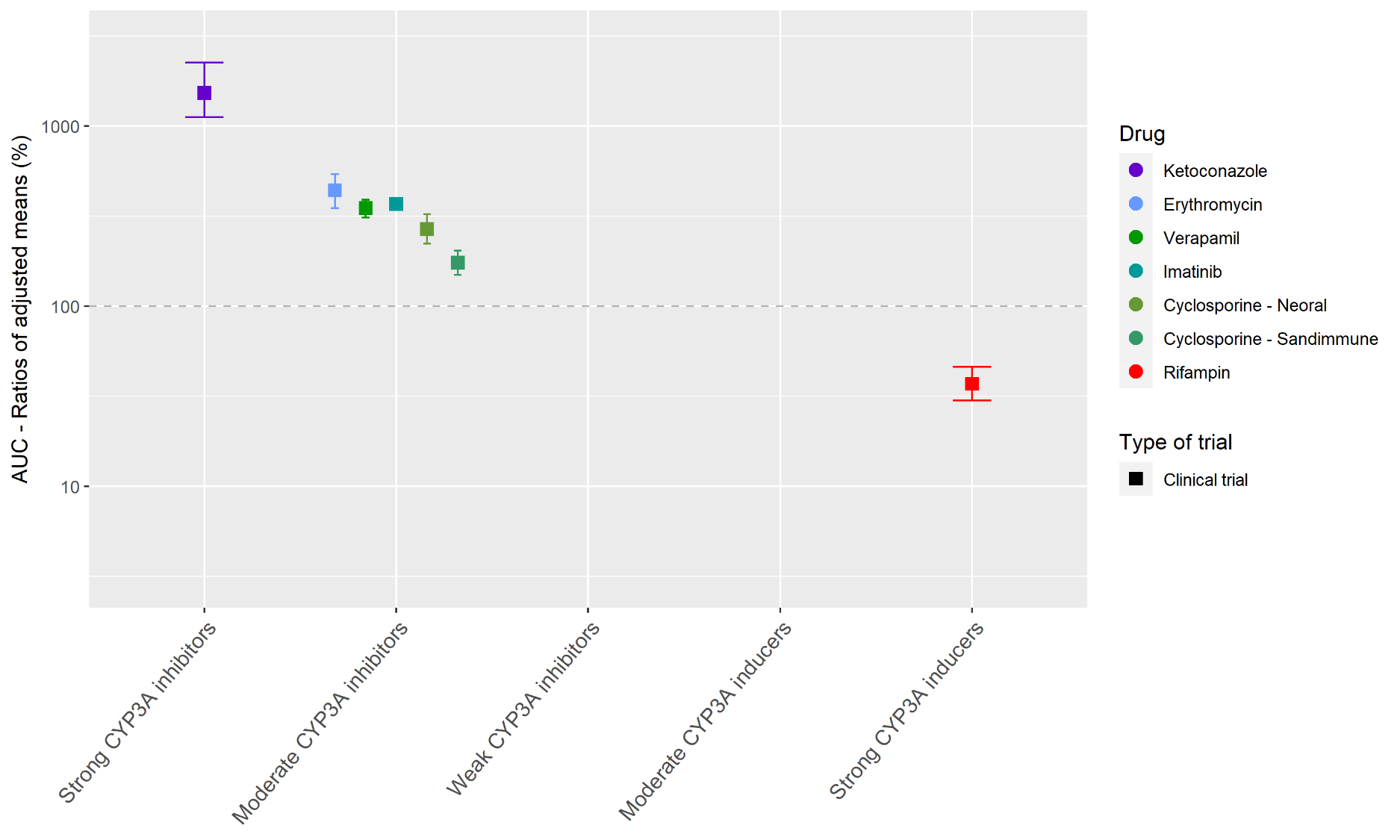
Supplemental Figure S2. Overview of the results from DDI studies of everolimus combined with CYP3A inhibitors and inducers. The colored symbols represent the increase or decrease in AUC caused by the interacting drug, expressed as adjusted mean ± 90% confidence interval (if available). The dashed line represents the baseline AUC (European Medicines Agency Committee for Medicinal Products For Human Use (CHMP); Kovarik et al., 2002a, 2002b, 2005b, 2005c, 2005a, 2006; Food and Drug Administration, 2008).


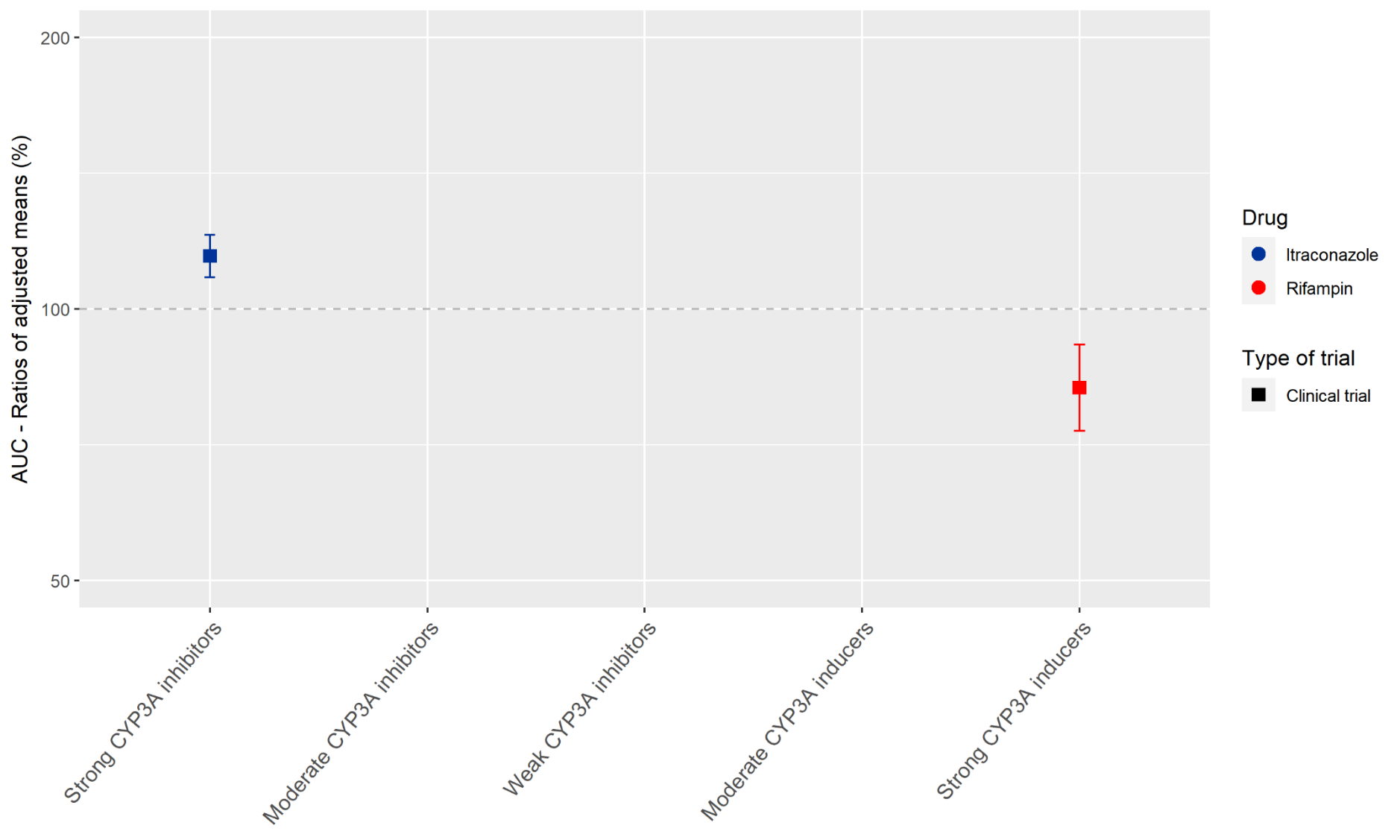


Supplemental figure S3. Overview of the results from DDI studies of lenvatinib combined with CYP3A inhibitors and inducers. The colored symbols represent the increase or decrease in AUC caused by the interacting drug, expressed as adjusted mean ± 90% confidence interval (if available). The dashed line represents the baseline AUC (Shumaker et al., 2014; Food and Drug Administration, 2015a; Shumaker et al., 2015).


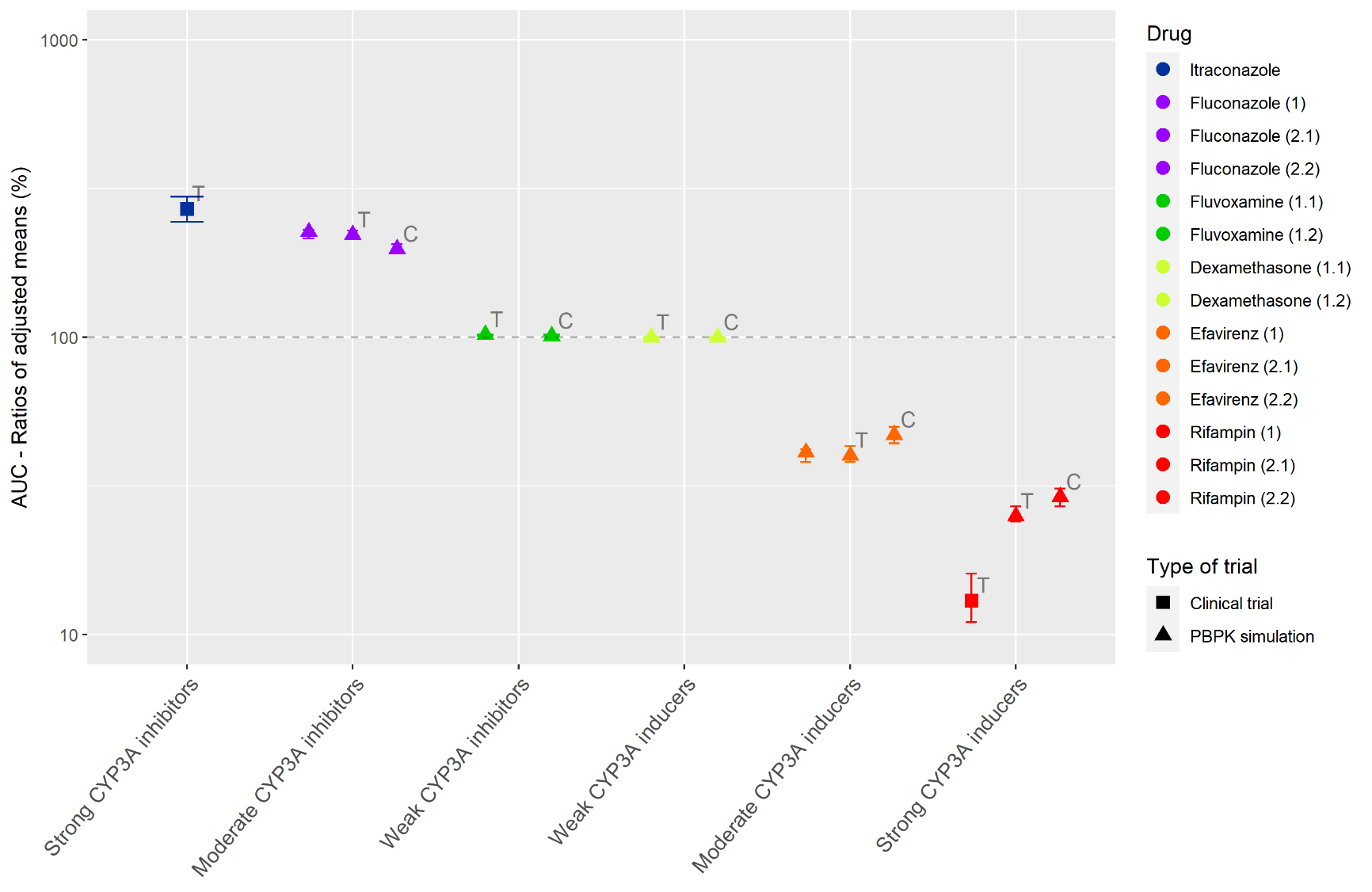


Supplemental figure S4. Overview of the results from DDI studies of olaparib combined with CYP3A inhibitors and inducers. The colored symbols represent the increase or decrease in AUC caused by the interacting drug, expressed as adjusted mean ± 90% confidence interval (if available). The dashed line represents the baseline AUC. T = DDI study with olaparib tablets; C = DDI study with olaparib capsules (Food and Drug Administration, 2014b; Dirix et al., 2016; Pilla Reddy et al., 2019).


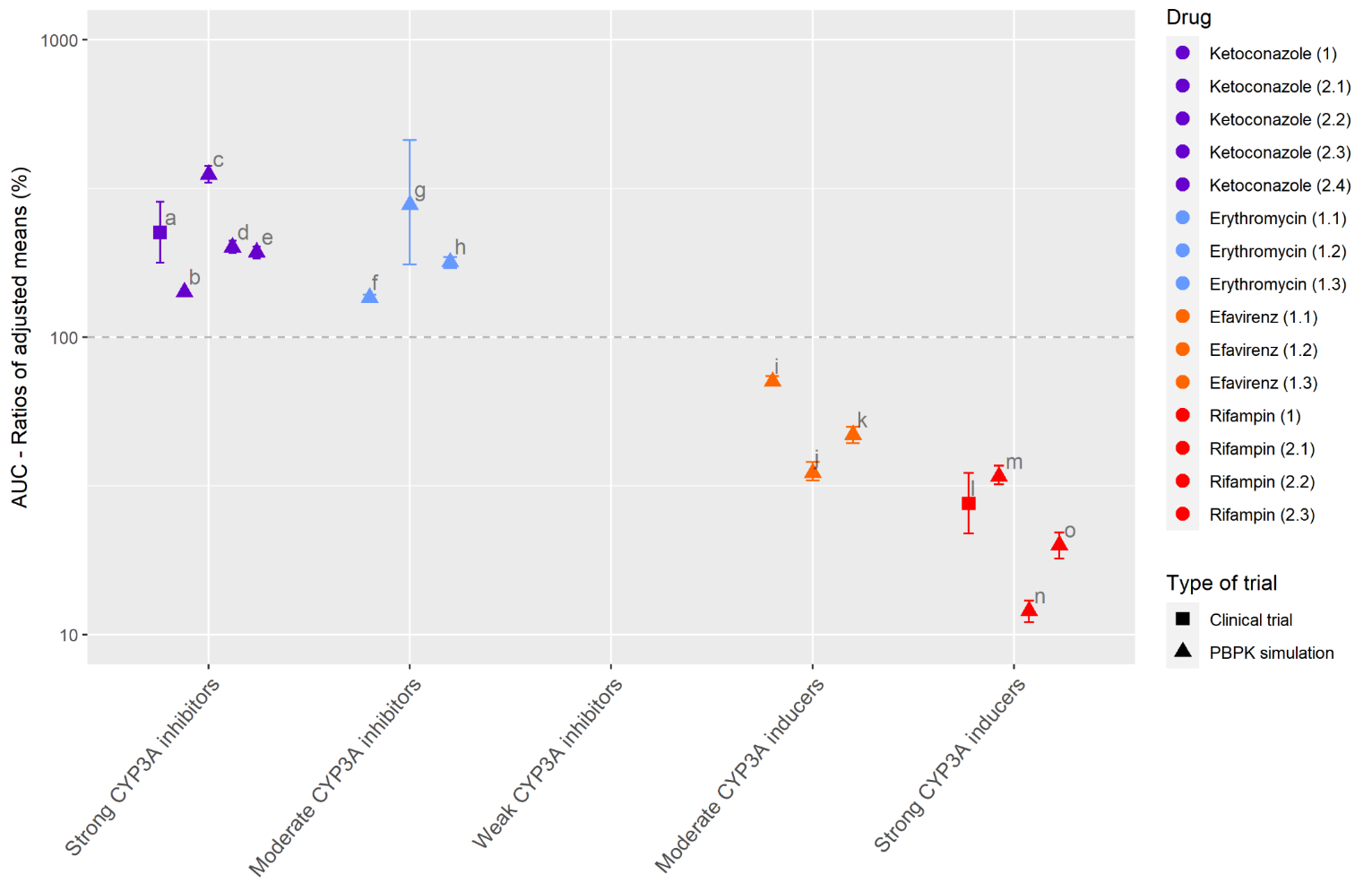


Supplemental figure S5. Overview of the results from DDI studies of sonidegib combined with CYP3A inhibitors and inducers. The colored symbols represent the increase or decrease in AUC caused by the interacting drug, expressed as adjusted mean ± 90% confidence interval (if available). The dashed line represents the baseline AUC (European Medicines Agency Committee for Medicinal Products For Human Use (CHMP), 2015; Food and Drug Administration, 2015b; Einolf et al., 2017).

a: sonidegib 800 mg single dose + ketoconazole 14 days in healthy subjects

b: sonidegib 200 mg single dose + ketoconazole 14 days in cancer patients

c: sonidegib 200 mg 120 days + ketoconazole 120 days in cancer patients

d: sonidegib 200 mg 133 days + ketoconazole 14 days in cancer patients

e: sonidegib 200 mg every other day 133 days + ketoconazole 14 days in cancer patients

f: sonidegib 200 mg single dose + erythromycin 14 days in cancer patients

g: sonidegib 200 mg 120 days + erythromycin 120 days in cancer patients

h: sonidegib 200 mg 133 days + erythromycin 14 days in cancer patients

i: sonidegib 200 mg single dose + efavirenz 14 days in cancer patients

j: sonidegib 200 mg 120 days + efavirenz 120 days in cancer patients

k: sonidegib 200 mg 133 days + efavirenz 14 days in cancer patients

l: sonidegib 800 mg single dose + rifampin 14 days in healthy subjects

m: sonidegib 200 mg single dose + rifampin 14 days in cancer patients

n: sonidegib 200 mg 120 days + rifampin 120 days in cancer patients

o: sonidegib 200 mg 133 days + rifampin 14 days in cancer patients

## *9.2 Figures for drugs with active metabolites*


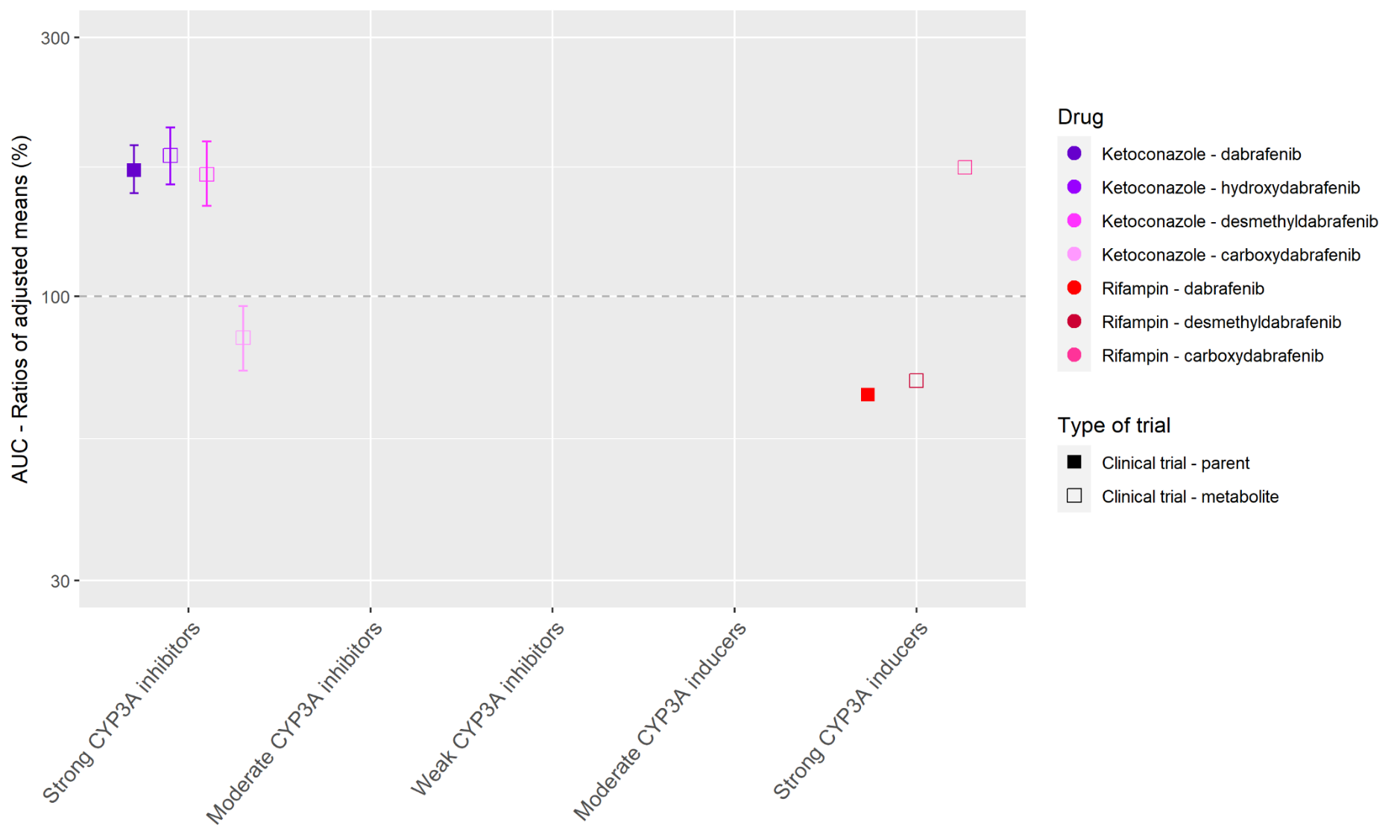


Supplemental figure S6. Overview of the results from DDI studies of dabrafenib combined with CYP3A inhibitors and inducers. The colored symbols represent the increase or decrease in AUC caused by the interacting drug, expressed as adjusted mean ± 90% confidence interval (if available). The dashed line represents the baseline AUC (Suttle et al., 2015; European Medicines Agency Committee for Medicinal Products For Human Use (CHMP), 2018).


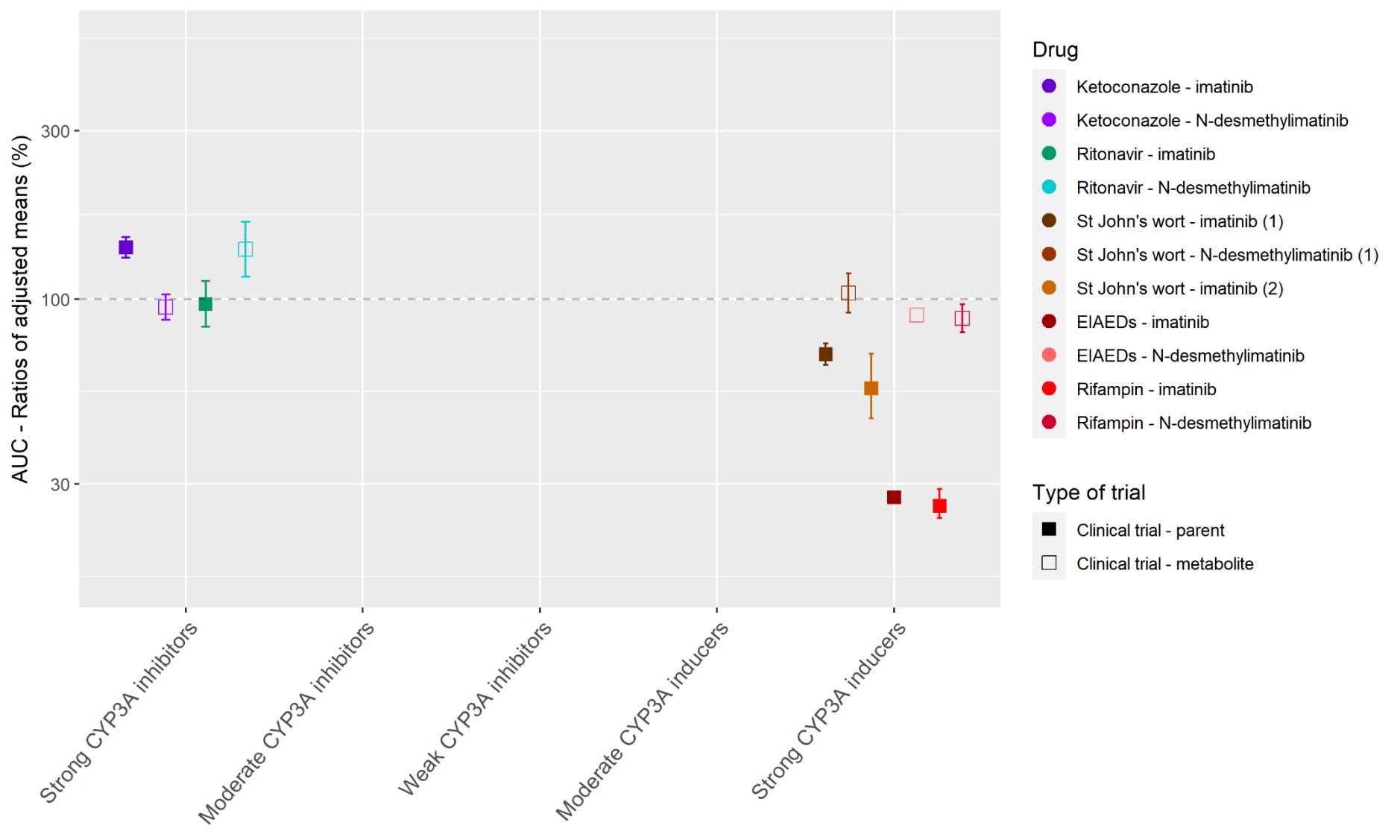


Supplemental figure S7. Overview of the results from DDI studies of imatinib combined with CYP3A inhibitors and inducers. The colored symbols represent the increase or decrease in AUC caused by the interacting drug, expressed as adjusted mean ± 90% confidence interval (if available). The dashed line represents the baseline AUC (Food and Drug Administration, 2001; Bolton et al., 2004; Frye et al., 2004; Smith et al., 2004; European Medicines Agency Committee for Medicinal Products For Human Use (CHMP), 2006; Wen et al., 2006; Van Erp et al., 2007).


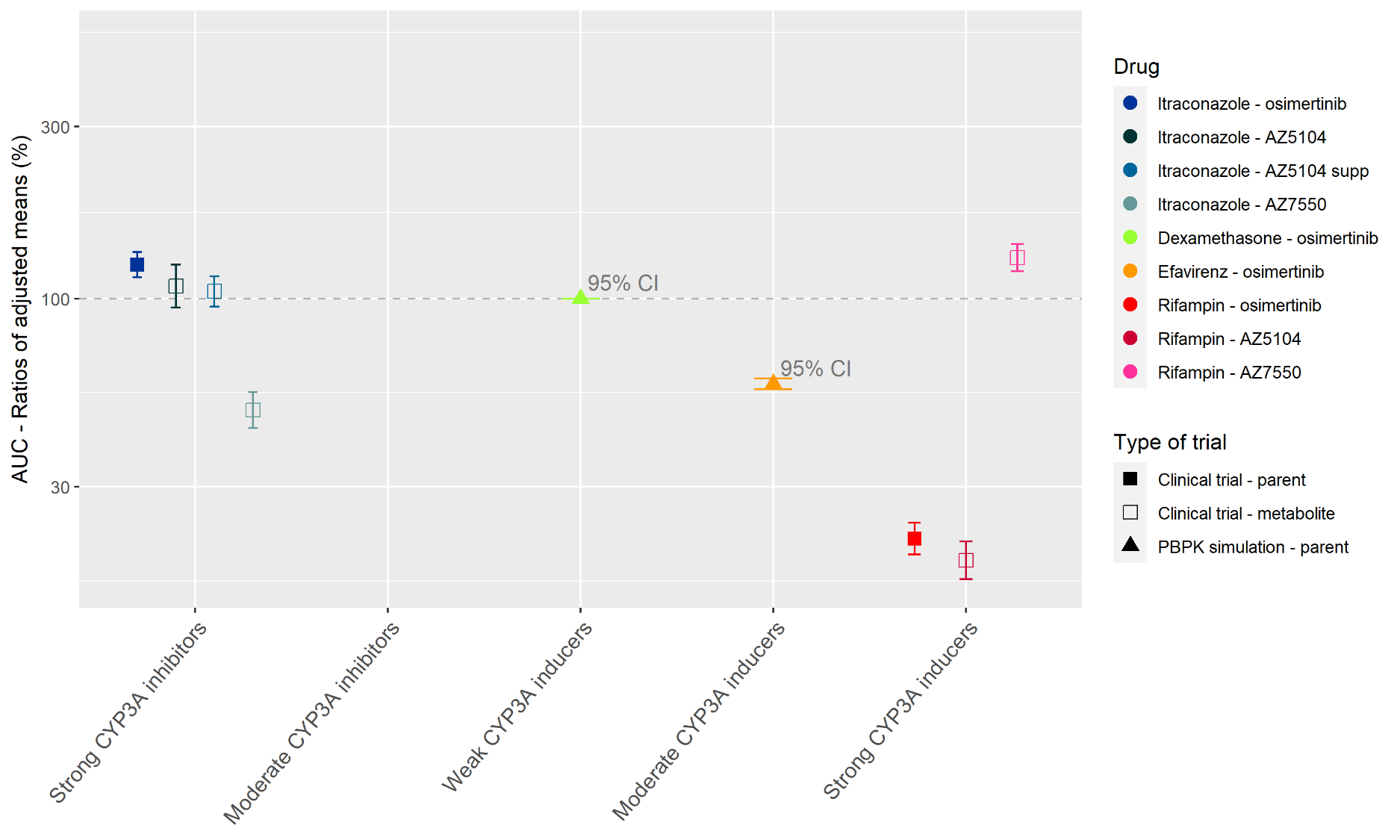


Supplemental figure S8. Overview of the results from DDI studies of osimertinib combined with CYP3A inhibitors and inducers. The colored symbols represent the increase or decrease in AUC caused by the interacting drug, expressed as adjusted mean ± 90% confidence interval (if available). The dashed line represents the baseline AUC. 95% CI = a 95% confidence interval is shown instead of a 90% confidence interval (European Medicines Agency Committee for Medicinal Products For Human Use (CHMP), 2016; Reddy et al., 2018; Vishwanathan et al., 2018).


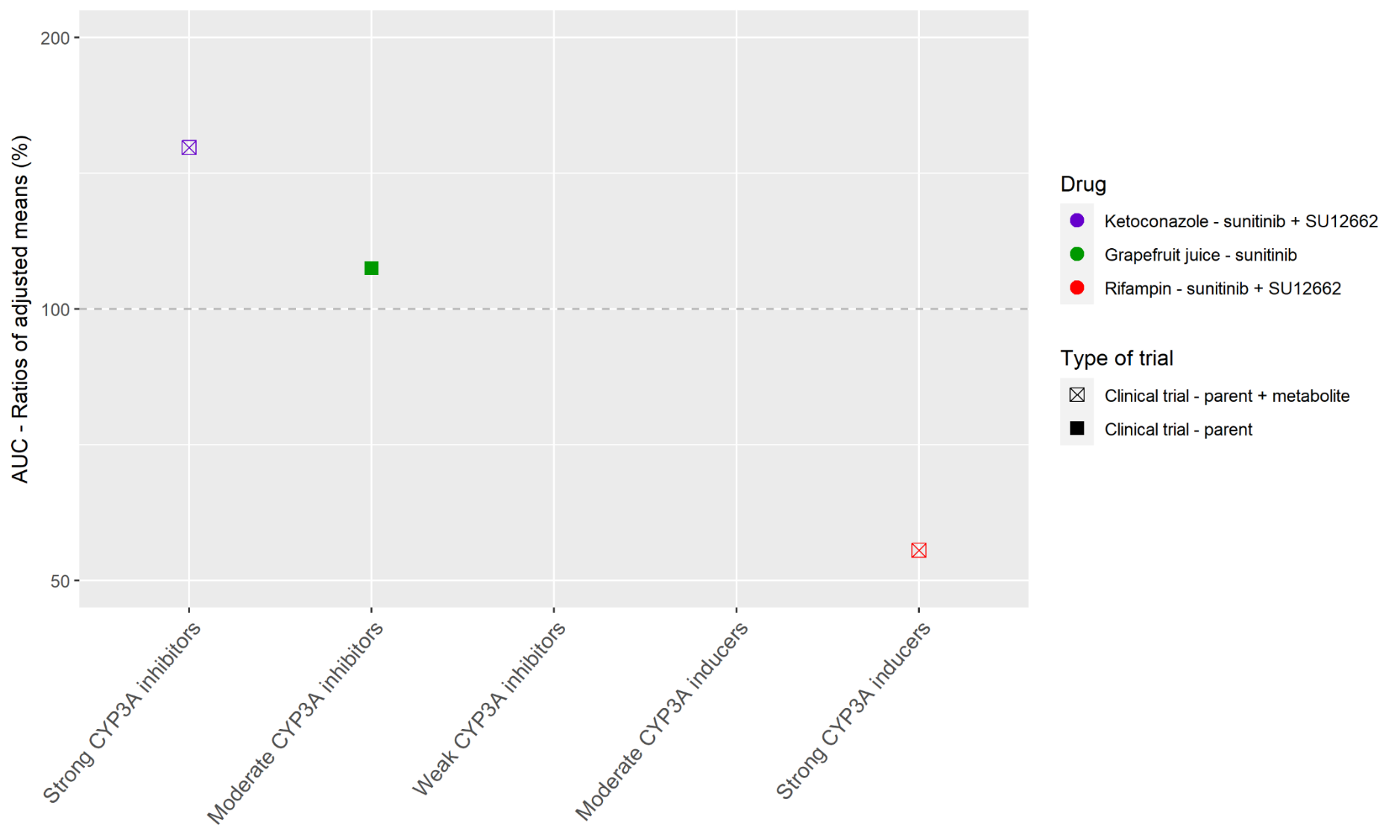


Supplemental figure S9. Overview of the results from DDI studies of sunitinib combined with CYP3A inhibitors and inducers. The colored symbols represent the increase or decrease in AUC caused by the interacting drug, expressed as adjusted mean. The dashed line represents the baseline AUC (Food and Drug Administration, 2005; Van Erp et al., 2011).
